# FDA Drug Repurposing Uncovers Modulators of Dopamine D_2_ Receptor Localization via Disruption of the NCS‑1 Interaction

**DOI:** 10.1021/acs.jmedchem.5c01626

**Published:** 2025-11-10

**Authors:** Daniel Muñoz-Reyes, Lorena Aguado, Sandra Arroyo-Urea, Carlos Requena, Sara Pérez-Suárez, Sonia Sánchez-Yepes, Josep Argerich, Celia Miró-Rodríguez, Eugenia Ulzurrun, Eulalia Rodríguez-Martín, Javier García-Nafría, Nuria E. Campillo, Alicia Mansilla, María José Sánchez-Barrena

**Affiliations:** † Department of Crystallography and Structural Biology, Institute of Physical-Chemistry “Blas Cabrera”, 16379 CSIC, Serrano 119, 28006 Madrid, Spain; ‡ Department of Neurobiology, Instituto Ramón y Cajal de Investigación Sanitaria, Hospital Universitario Ramón y Cajal, 28034 Madrid, Spain; § Institute for Biocomputation and Physics of Complex Systems (BIFI) and Laboratorio de Microscopías Avanzadas (LMA), 537113 University of Zaragoza, 50018 Zaragoza, Spain; ∥ 54446 Centro de Investigaciones Biológicas Margarita Salas - CSIC, C/Ramiro de Maeztu 9, 28040 Madrid, Spain; ⊥ Instituto de Ciencias Matemáticas-CSIC, C/Nicolas Cabrera 13-15, 28049 Madrid, Spain

## Abstract

Dopamine D_2_ receptor (D_2_R) regulates key aspects of motor control, cognition, and reward. Its function depends not only on ligand binding and signaling efficacy but also on the dynamic control of receptor localization at the cell surface. Neuronal calcium sensor 1 (NCS-1) interacts with D_2_R in a Ca^2+^-dependent manner. Using in vitro and cellular assays, we found that NCS-1 promotes D_2_R trafficking to the plasma membrane through active exocytosis while preserving canonical receptor pharmacology. A screen of FDA-approved drugs identified protein–protein interaction (PPI) modulators targeting the NCS-1/D_2_R interface. Azilsartan medoxomil, atorvastatin, and vilazodone disrupt this interaction, reducing D_2_R surface expression. Structural studies revealed that these compounds target NCS-1, overlap the D_2_R binding site, and perturb the dynamics of the regulatory helix H10. These findings reveal an unexploited intracellular mechanism to modulate D_2_R function via PPI modulation, offering a novel strategy to fine-tune dopaminergic tone beyond receptor blockade or direct agonism.

## Graphical abstract

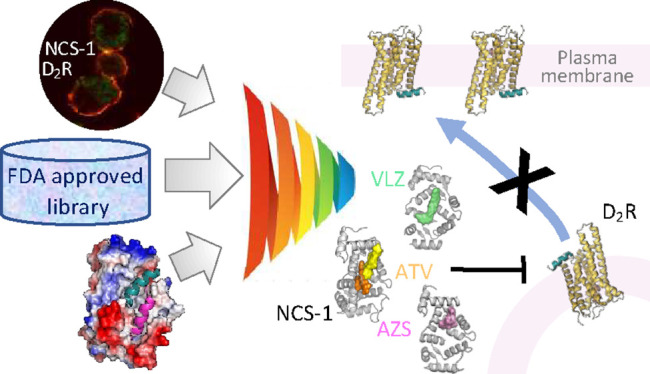

## Introduction

The dopamine D_2_ receptor (D_2_R) is a member of the G protein–coupled receptor (GPCR) family and transduces signals primarily through the coupling and activation of heterotrimeric Gαβγ proteins (G_i/O_ type) and β-arrestins.^1,2^ D_2_R is a key pharmacological target in the treatment of various neuropsychiatric disorders, including Tourette’s syndrome, schizophrenia, Parkinson’s disease and bipolar disorders, conditions commonly associated with imbalance in dopaminergic transmission. This is underscored by the fact that D_2_-like receptors are the major target for drugs in the treatment of Parkinson’s disease (agonists) and the majority of antipsychotic and neuroleptic drugs (antagonists and partial agonists), with D_2_R representing the predominant isoform expressed in the brain. In addition, human genetic variants in the D_2_R have been shown to yield motor, cognitive, and neuropsychiatric deficits.^3–10^

The density of postsynaptic dopamine D_2_ receptors critically determines neuronal sensitivity to dopamine, thereby influencing both the intensity and duration of dopaminergic signaling. Proper neural communication relies on the maintenance of an optimal level of D_2_R surface expression: insufficient receptor availability may attenuate dopamine-mediated responses, whereas excessive expression can result in heightened sensitivity and dysregulated signaling activity. In addition to their postsynaptic role, D_2_Rs also function as presynaptic autoreceptors located on dopaminergic neurons. Activation of these autoreceptors initiates intracellular signaling pathways that inhibit further dopamine release, establishing a negative feedback loop essential for maintaining dopaminergic homeostasis and preventing neurotransmitter overaccumulation.^11,12^

The functional availability of D_2_Rs at both pre- and postsynaptic sites is tightly regulated by receptor trafficking processes, including insertion into the membrane, internalization, and recycling. These dynamic mechanisms control the number of receptors accessible at the cell surface and are therefore central to dopaminergic regulation.^13^ Among the proteins implicated in modulating D_2_R trafficking is Neuronal Calcium Sensor 1 (NCS-1), which has been shown to influence receptor localization, although the precise molecular mechanisms remain incompletely defined.^14^ Elucidating these regulatory pathways is critical for understanding dopamine-related neural function and its dysregulation in neuropsychiatric disorders.

NCS-1 is a high-affinity calcium binding protein that plays diverse roles in the nervous system, including synaptic transmission and plasticity, due to its ability to interact and regulate multiple protein targets. At the molecular level, NCS-1 contains two pairs of EF-hands and a C-terminal dynamic helical region that inserts into a surface exposed hydrophobic groove and acts as a ligand mimic in the absence of a binding partner.^15^ NCS-1 regulates the activity of Ca^2+^ channels such as the inositol 1,4,5-trisphosphate receptors (InsP_3_Rs) and several proteins implicated in G-protein signaling: dopamine D_2_ receptor, GRK2 kinase or the Gα chaperone and GEF Ric-8A, through both in a Ca^2+^- dependent and -independent mechanisms.^14,16,17^ Dysregulation of NCS-1 has been associated with several neuropathological processes including X-linked intellectual disability, autism, schizophrenia, and bipolar disorder.^18–21^ Notably, elevated NCS-1 expression has been reported in the prefrontal cortex of individuals with schizophrenia and bipolar disorder.^19^ Similarly, an increase in postsynaptic D_2_R levels has been observed in antipsychotic-free schizophrenia patients. Collectively, these findings highlight the importance of investigating the regulatory role of NCS-1 in D_2_R function and its impact on cellular localization, receptor signaling and pharmacology.

Previous studies have identified NCS-1 as a potential therapeutic target, where modulation of the NCS-1/Ric-8A interaction by small molecules can restore synaptic function, showing promise in models of fragile X syndrome and Alzheimer’s disease.^22–24^ These compounds exert their effects by modulating the conformational dynamics of the C-terminal helix H10 of NCS-1, which serves as a regulatory gatekeeper for Ric-8A binding, which inserts a long helix into an elongated and hydrophobic cavity under resting intracellular Ca^2+^ state.^17,22^ In contrast, NCS-1 interacts with the cytosolic C-terminal helix H8 of the dopamine D_2_ receptor under elevated intracellular Ca^2+^ concentrations. Crystallographic studies of NCS-1 in complex with a peptide containing this intracellular region of the receptor have shown that the helix H10 inserts into the elongated crevice, reshaping NCS-1 molecular surface to create the D_2_R recognition interface, where two intracellular H8 helices bind to (Figure 1).^14,25,26^

**Figure 1:**
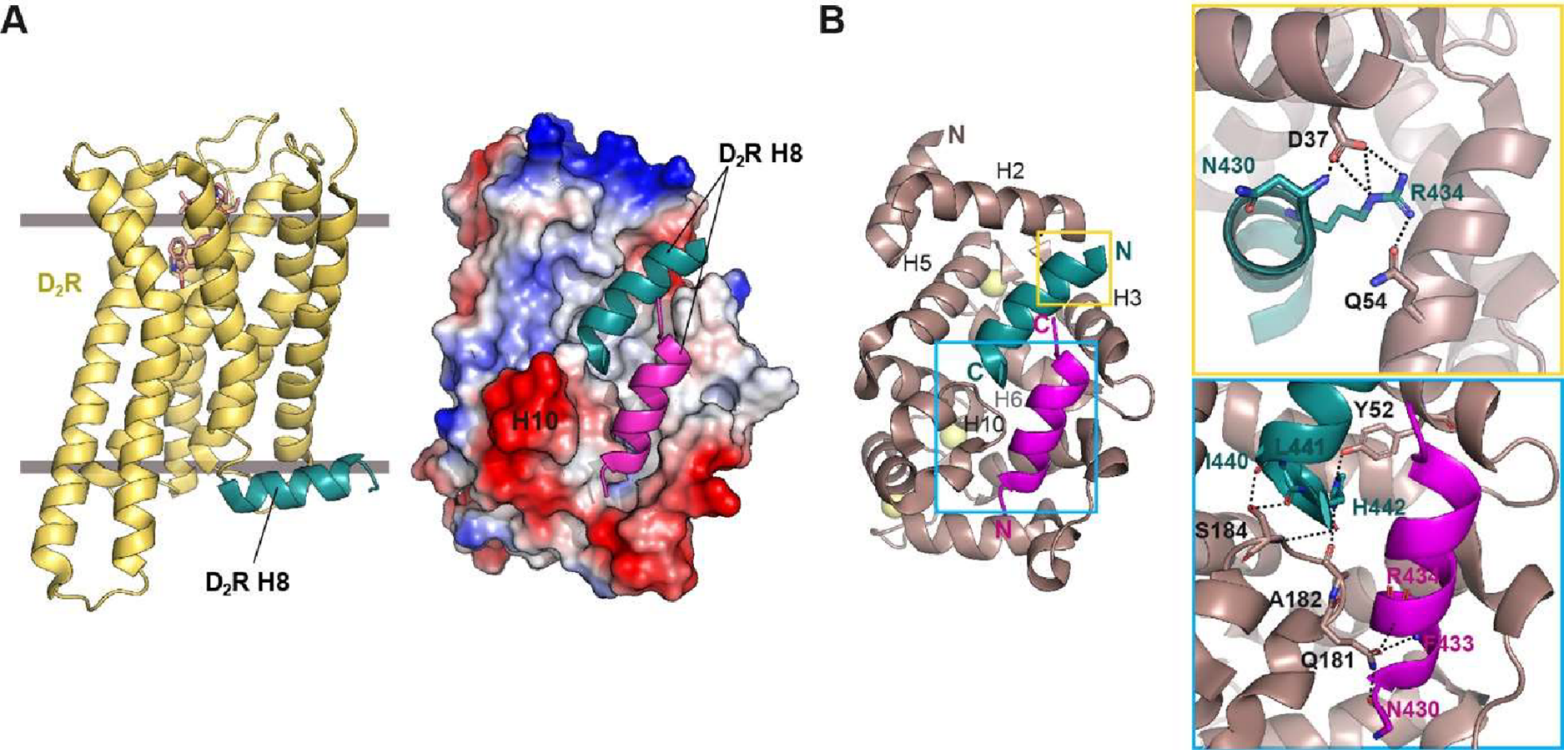
NCS-1 helix H10 participates in dopamine D_2_ receptor recognition. (A) Left: Structure of D_2_R bound to an agonist (PDB: 6VMS,^27^), with the intracellular helix H8 shown in green. Right: Molecular surface representation of NCS-1 bound to peptides containing the D_2_R cytosolic helix H8 (PDB: 5AER,^26^). (B) Details of the strong polar interactions occurring at the ends of the H8 helices. Side chains not involved in H-bond interactions with NCS-1 are not shown.

Building on these structural insights, we implemented a structure-based drug repurposing approach using an FDA-approved compound library to identify small-molecule modulators of the NCS-1/D_2_R interaction. These compounds serve not only as molecular probes to investigate the role of NCS-1 in D_2_R trafficking and signaling, but also as potential therapeutic hits for the treatment of neuropsychiatric disorders such as schizophrenia and bipolar disorder.

## Results

### NCS-1 Regulates the Subcellular Localization of D_2_R

To assess the effects of NCS-1 on D_2_R localization, cells were transfected with plasmids encoding D2R, either alone or co-transfected with NCS-1. Sequential immunostaining was performed to detect membrane-bound D_2_R in red and cytoplasmic D_2_R in green. Quantification of subcellular localization based on the confocal images showed that under basal conditions, D_2_R is mainly localized in the cytoplasm, whereas NCS-1 overexpression promotes a significant accumulation of D_2_R at the plasma membrane (Figure 2A,C). Consistently, we observed that this effect scales with increasing NCS-1 levels, further supporting a dose-dependent relationship between NCS-1 abundance and D_2_R membrane localization (Supplementary Figure 1A).

**Figure 2:**
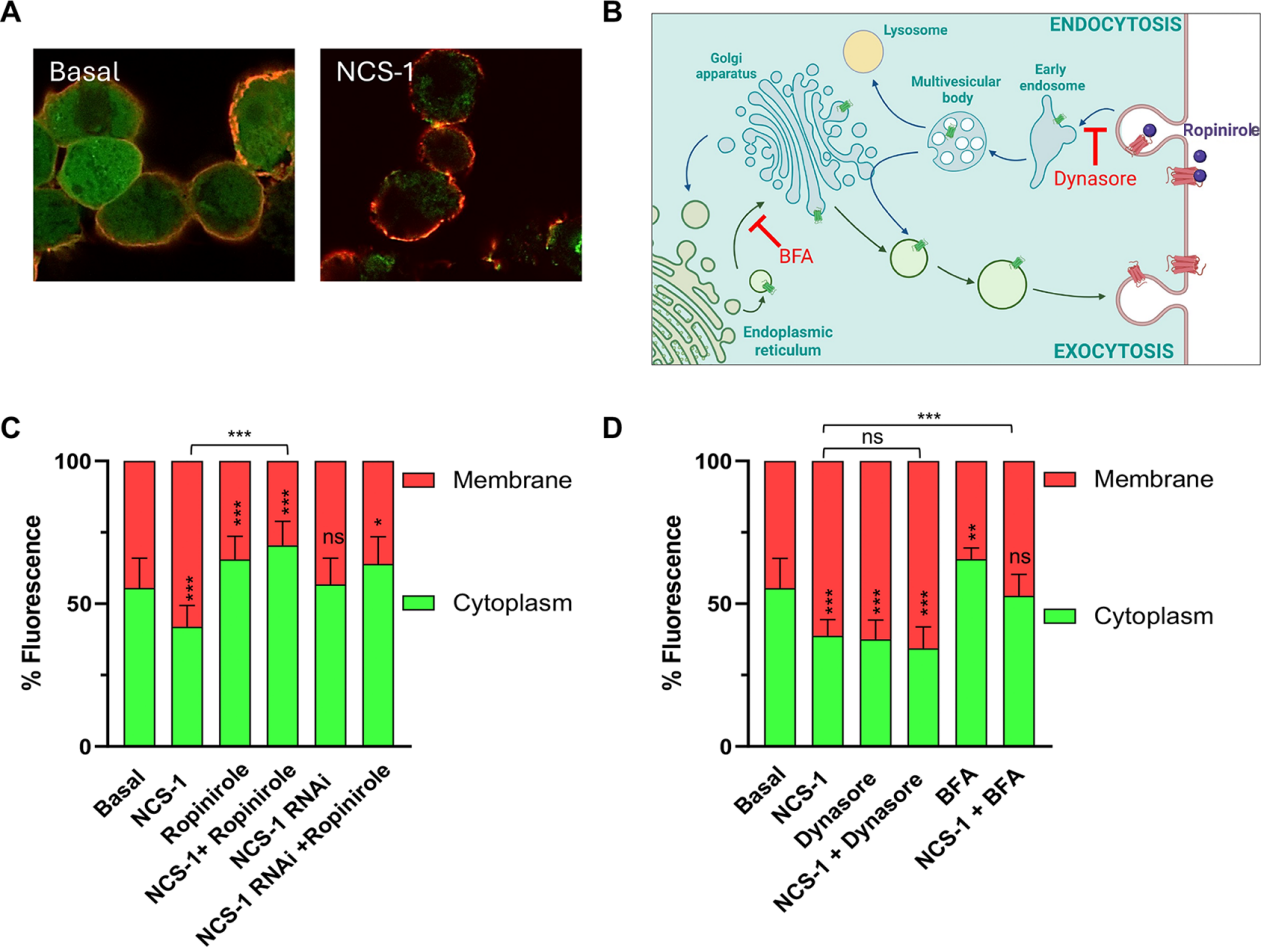
Role of NCS-1 in the subcellular localization of D_2_R. (A) Representative confocal images of HEK293 cells transfected with D_2_R alone (basal) or cotransfected with D_2_R and NCS-1 (NCS-1). D_2_R is FLAG-tagged, and a sequential immunofluorescence protocol was used to differentially label plasma-membrane-localized D_2_R (red) and intracellular D_2_R (green). (B) Schematic diagram illustrating D_2_R trafficking via endocytic and exocytic pathways, along with the targets of the pharmacological agents used in the study: Ropinirole (a D_2_R agonist that promotes receptor internalization), Dynasore (an endocytosis inhibitor), and Brefeldin A (BFA, an exocytosis inhibitor). Illustration created with BioRender.com. (C) Quantification of D_2_R fluorescence distribution between membrane (red) and cytoplasm (green) in HEK293 cells transfected with D_2_R alone or with NCS-1 or NCS-1 RNAi. Ropinirole is used as a D_2_R agonist. (D) Effect of Dynasore and BFA on NCS-1-mediated D_2_R redistribution. Data are expressed as the percentage of total D_2_R fluorescence localized at the membrane (red) and in the cytoplasm (green). Asterisks within bars indicate statistical comparison with the basal condition (D_2_R alone), while horizontal bars above the graphs indicate comparisons between specific experimental conditions. Statistical significance: ns = not significant, *p < 0.05, **p < 0.01, ***p < 0.001.

Ropinirole, a D_2_R agonist known to trigger internalization, reversed this effect when applied to NCS-1-overexpressing cells, suggesting that NCS-1 promotes D_2_R membrane stabilization, which can be counteracted by agonist-induced endocytosis. Interestingly, knockdown of HEK293 endogenous NCS-1 expression via RNA interference led to a shift of D_2_R back to the cytoplasm, further supporting the role of NCS-1 in plasma membrane localization (Figure 2B,C).

To dissect the underlying trafficking mechanisms, we used Dynasore (endocytosis inhibitor) and Brefeldin A (BFA, exocytosis inhibitor) in combination with NCS-1 overexpression (Figure 2B,D).^28^ Dynasore did not significantly affect NCS-1-induced membrane localization of D_2_R, suggesting that NCS-1 does not act primarily by blocking endocytosis. In contrast, BFA treatment prevented the NCS-1-induced increase in membrane D_2_R, indicating that NCS-1 enhances D_2_R membrane localization through a BFA-sensitive exocytic pathway.

Overall, these findings indicate that NCS-1 promotes the trafficking of D_2_R to the plasma membrane through a mechanism dependent on active exocytosis rather than inhibition of endocytosis. This highlights NCS-1 as a positive regulator of D_2_R surface expression and may have implications for dopaminergic signaling regulation.

### Role of NCS-1 in Regulating Dopamine D_2_ Receptor Function

The dopamine D_2_ receptor belongs to the GPCR family and hence signals by coupling to and activating heterotrimeric Gαβγ proteins and β-arrestins.^2^ It is currently unclear whether binding of NCS-1 at the intracellular helix H8 affects the receptor pharmacological properties, since its binding potentially poses a steric conflict with intracellular transducers (Figure 1). For this purpose, we performed signaling assays in HEK293T cells, titrating quinpirole, a widely used D_2_R agonist, to obtain potency (pEC_50_) and efficacy (*E*_max_) pharmacological values in three different assays that included Gαβγ activation (using BRET2 assays),^29^ MiniGα recruitment (using Nanobit assays)^30^ and β-arrestin recruitment to the receptor (using split Nanoluc assays),^31^ all in the presence and absence of cotransfected NCS-1. Overall, the potency of quinpirole in the three assays was virtually identical in the absence and presence of NCS-1 (Figure 3 and Supplementary Figure 2), highlighting that NCS-1 does not have an impact in the canonical functions of the receptor. Additionally, efficacy was also identical when monitoring Gαβγ activation and the recruitment of β-arrestin (Figure 3), hence also independent of the presence of NCS-1. These signaling assays monitor signaling processes immediately at the receptor, lacking signal amplification and thus are not sensitive to minor changes in receptor expression.^29^ Therefore, we do not expect to see a difference in efficacy due to the increase in surface expression of the D_2_R caused by NCS-1 cotransfection. An increase in efficacy was seen in the Nanobit assay upon NCS-1 cotransfection (Supplementary Figure 2), however it seems a much larger efficacy effect than what would be expected from the increase in surface expression seen with confocal microscopy (Figure 2). Although we cannot fully explain the increase in efficacy on these assays, MiniGα proteins have been shown to disrupt receptor endocytic trafficking,^32^ which could, together with NCS-1, yield complex trafficking effects, only observed when using such protein chimeras but not when using active (nondominant negative) Gαβγ proteins and β-arrestins. Overall, we conclude that overexpressing NCS-1 does not have an impact on the pharmacological profile of the D_2_R in this cellular model system.

**Figure 3:**
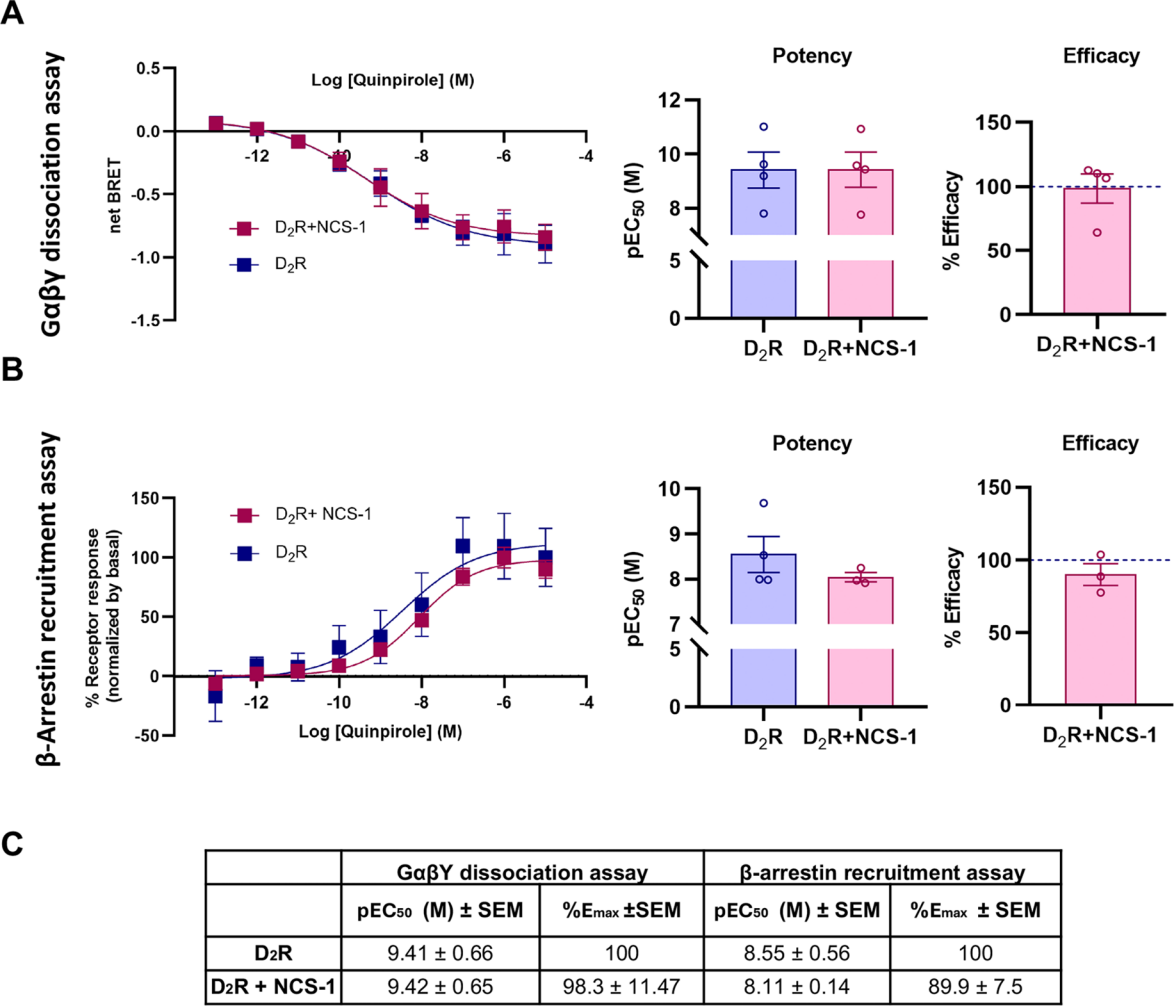
Effect of NCS-1 on D_2_-receptor-mediated G protein activation and β-arrestin recruitment. (A) Concentration–response curves and bar plots of D_2_R (blue) and D_2_R cotransfected with NCS-1 (pink) upon G_OA_ activation with quinpirole using cellular BRET2 assays. (B) Concentration–response curves and bar plots of D_2_R (blue) and D_2_R cotransfected with NCS-1 (pink) upon β-arrestin recruitment with quinpirole. (C) Summary table with pEC_50_ and *E*_max_ values derived from the Gαβγ dissociation and β-arrestin recruitment assays. Data are presented as means ± SEM; pEC_50_ and *E*_max_ values of all data are derived from four independent experiments performed in technical triplicates, except for D_2_R + NCS-1 in β-arrestin recruitment assay (*n* = 3). *E*_max_ bar plots are displayed as normalized efficacy to D_2_R (dashed blue line).

### Virtual Screening to Find FDA-Approved Drugs Targeting the NCS-1/D_2_R Interface

To identify compounds capable of modulating the NCS-1/D_2_R protein–protein interaction (PPI) interface, virtual screenings were conducted using the FDA-approved drug library comprising 2143 compounds. Given the structural characteristics of NCS-1, particularly the key role of helix 10 (H10) in mediating interactions with other proteins,^22,23,33^ we proposed conducting the virtual screening using different structural models of NCS-1. To this end, a structural analysis was performed to select the most suitable NCS-1 template models for screenings.

NCS-1 contains a dynamic C-terminal helix (H10) that shapes the exposed hydrophobic crevice, the primary binding site for target proteins.^16,17,22,26^ The crystal structure of NCS-1 in complex with a peptide containing the cytosolic C-terminal H8 helix of D_2_R (PDB ID: 5AER,^26^) revealed that H10 defines two cavities: an upper cavity where a D_2_R H8 is deeply inserted and a lower cavity where a second copy of the D_2_R H8 is found. In addition, NCS-1 H10 directly interacts with D_2_R H8, with residues D37, Y52 and Q181 establishing strong interactions and thus, playing key roles in receptor recognition (Figure 1). Given the importance of NCS-1 H10 in D_2_R binding, we selected two NCS-1 structural templates for virtual screenings: one with H10 inserted in the crevice (PDB ID: 5AAN,^22^) and another with H10 exposed (PDB ID: 6QI4,^23^). Furthermore, a filtering criterion was applied to prioritize molecules interacting with D37, Y52 and Q181.

A hierarchical virtual screening approach was employed, targeting the two proposed structural models of NCS-1. Both targets were subjected to a three-staged virtual screening protocol consisting of a preliminary docking study by using the SP Glide docking algorithm, a second docking by applying the XP Glide docking algorithms and a final rescoring by applying the Prime MM-GBSA method. For each screened target, at least the 20 best ranked FDA drugs according to the MM-GBSA score were preliminarily selected. Finally, the compounds were filtered based on their interactions with key residues (D37, Y52 and Q181) in order to identify molecules with the most favorable interactions (Figure 4 and Supplementary Table 1).

**Figure 4:**
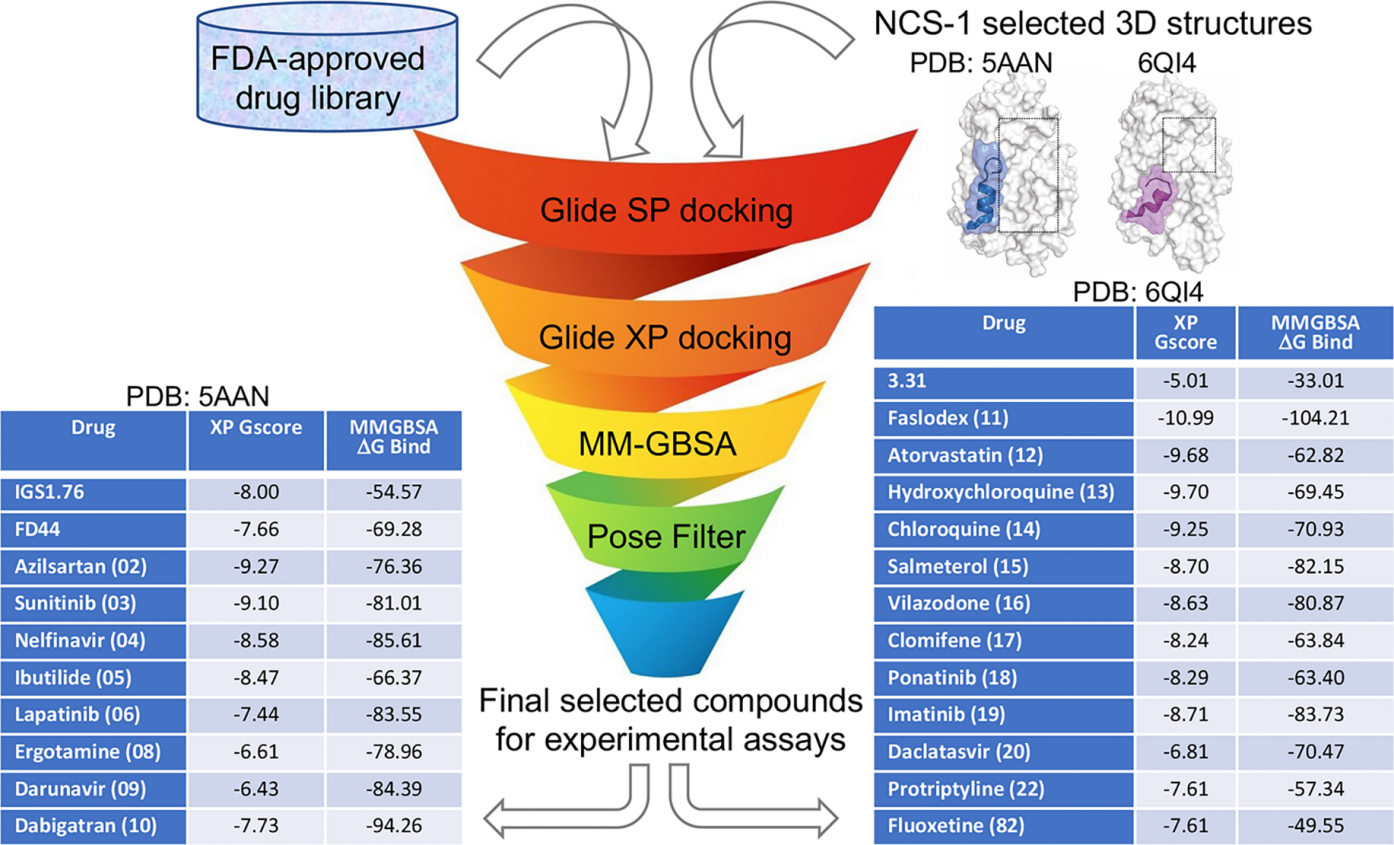
Virtual screening-selected FDA-approved compounds. Score units of XP and MM-GBSA is kcal/mol. In parentheses, the FDA code given for each compound is shown. Control compounds are shown: IGS1.76, FD44, and 3.31 (also know as 3b).^22,23,33^

Additional filters were applied to prioritize molecules with sufficient hydrophobic character to ensure blood brain barrier permeability. Among them, compounds intended for a veterinary and/or cosmetic use, biocides, laxative or topical-administered drugs were not considered for experimental assays. Ultimately, 20 hit molecules were selected for experimental evaluation (Figure 4).

### Binding of Candidate FDA-Approved Drugs to NCS-1

To experimentally assess the binding of the FDA-approved drug library to NCS-1 we used tryptophan emission fluorescence.^22,23,33^ Fluorescence intensity changes were measured at 330 nm in full-length NCS-1 upon increasing concentrations of the selected compounds (Figure 5A). Compounds exhibiting fluorescence emission within the protein’s emission range (azilsartan medoxomil (FDA02), darunavir (FDA09), faslodex (FDA11), salmeterol (FDA15), ziprasidone (FDA21) and protriptyline (FDA22)) were further analyzed using surface plasmon resonance (SPR) (Figure 5B). In these experiments, full-length NCS-1 was cross-linked to the surface of the biosensor, through its lysine amino acids, since these residues are concentrated on the upper surface and opposite to the hydrophobic crevice. This arrangement ensured that the hydrophobic crevice remained exposed to the solvent.

**Figure 5:**
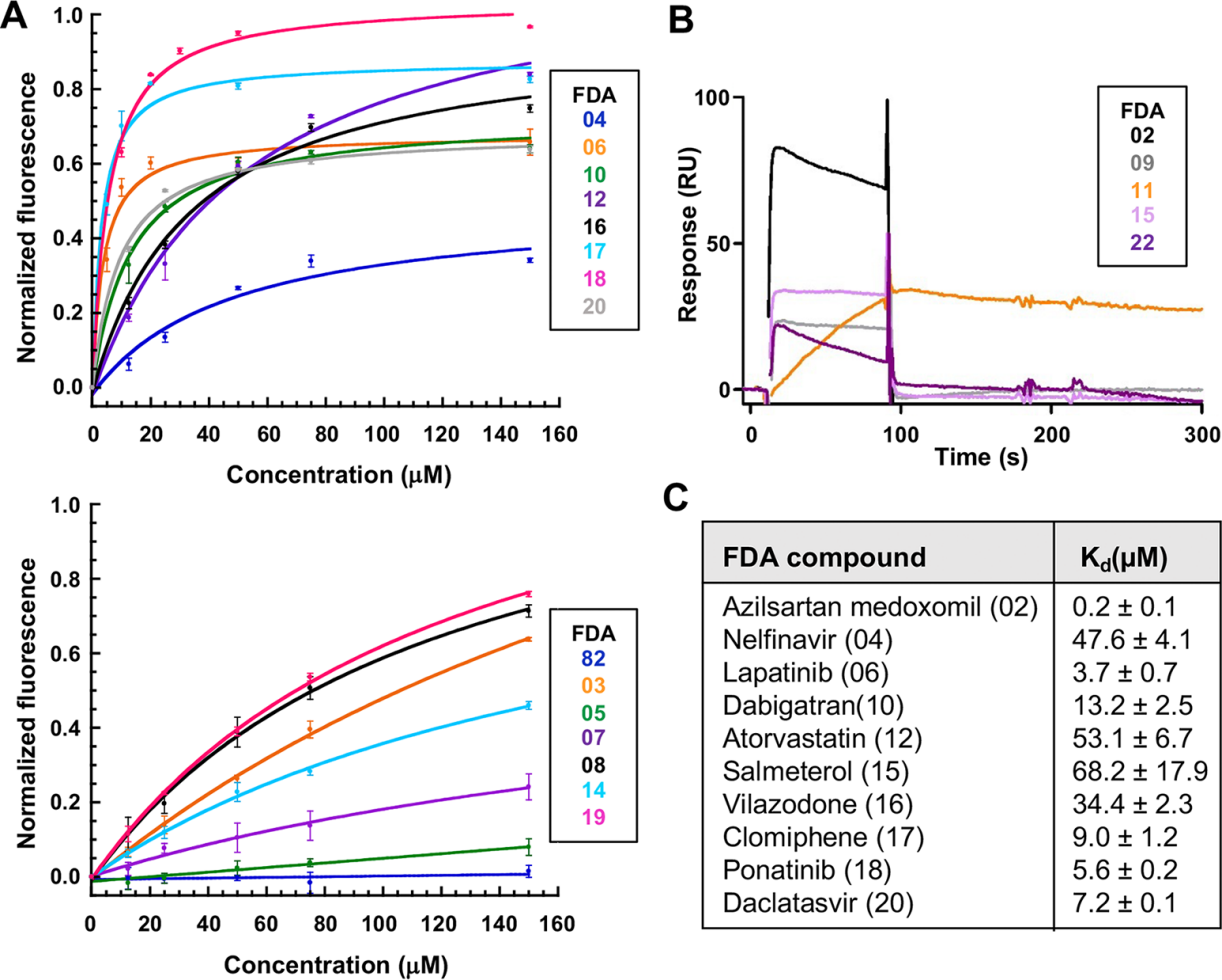
Binding of FDA hits to NCS-1: (A) Trp emission fluorescence assays. The upper graph displays compounds with affinities below 100 μM, while the lower graph shows those with affinities above 100 μM. (B) Surface plasmon resonance experiments. Binding of the tested compounds at 100 μM over CM4-immobilized NCS-1. Each compound is indicated with a different color code. (C) Binding affinities of the best FDA-selected compounds. Dissociation constants (*K*_d_ ± SD) are indicated. Only compounds with a *K*_d_ below 100 μM are shown. In parentheses, the FDA code given for each compound is shown.

Due to the low solubility of ziprasidone (FDA21) under the experimental conditions, it was not possible to obtain affinity data and the compound was discarded. Compounds that failed to bind NCS-1 or exhibited a dissociation constant (*K*_d_) above 100 μM were excluded from further analysis. This group included fluoxetine (FDA82), sunitinib (FDA03), ibutilide (FDA05), capastat (FDA07), ergotamine (FDA08), hydroxychloroquine (FDA13), chloroquine (FDA14), imatinib (FDA19), and protriptyline (FDA22) (Figure 5). In contrast, compounds with affinities below 100 μM, including azilsartan medoxomil (FDA02), nelfinavir (FDA04), lapatinib (FDA06), dabigatran etexilate (FDA10), atorvastatin (FDA12), salmeterol (FDA15), vilazodone (FDA16), clomiphene (FDA17), ponatinib (FDA18) and daclatasvir (FDA20), were selected for further evaluation or their potential to modulate protein–protein interactions (Figure 5C).

### PPI Modulatory Activity of the FDA-Approved Drugs

To identify the FDA-approved compounds capable of disrupting the interaction in physiological conditions we have used the Proximity Ligation Assay (PLA) technology, that allows the evaluation of protein–protein interactions in situ with high sensitivity.^34^ This tool is based on the specific antibody recognition of the two proteins of interest and takes advantage of DNA primers covalently linked to the secondary antibodies. Only when the complex is formed, probe primers bind, amplification occurs, and fluorescent detection is possible.

The extent of NCS-1/D_2_R interaction was quantified after 16 h of treatment with 5 μM of the selected FDA-approved compounds. Data are normalized to vehicle-treated cells (set to 100%). Only three compounds: azilsartan medoxomil (FDA02), atorvastatin (FDA12) and vilazodone (FDA16) had the majority of values below the 100% threshold, highlighting their modulatory potential (Figure 6A).

**Figure 6:**
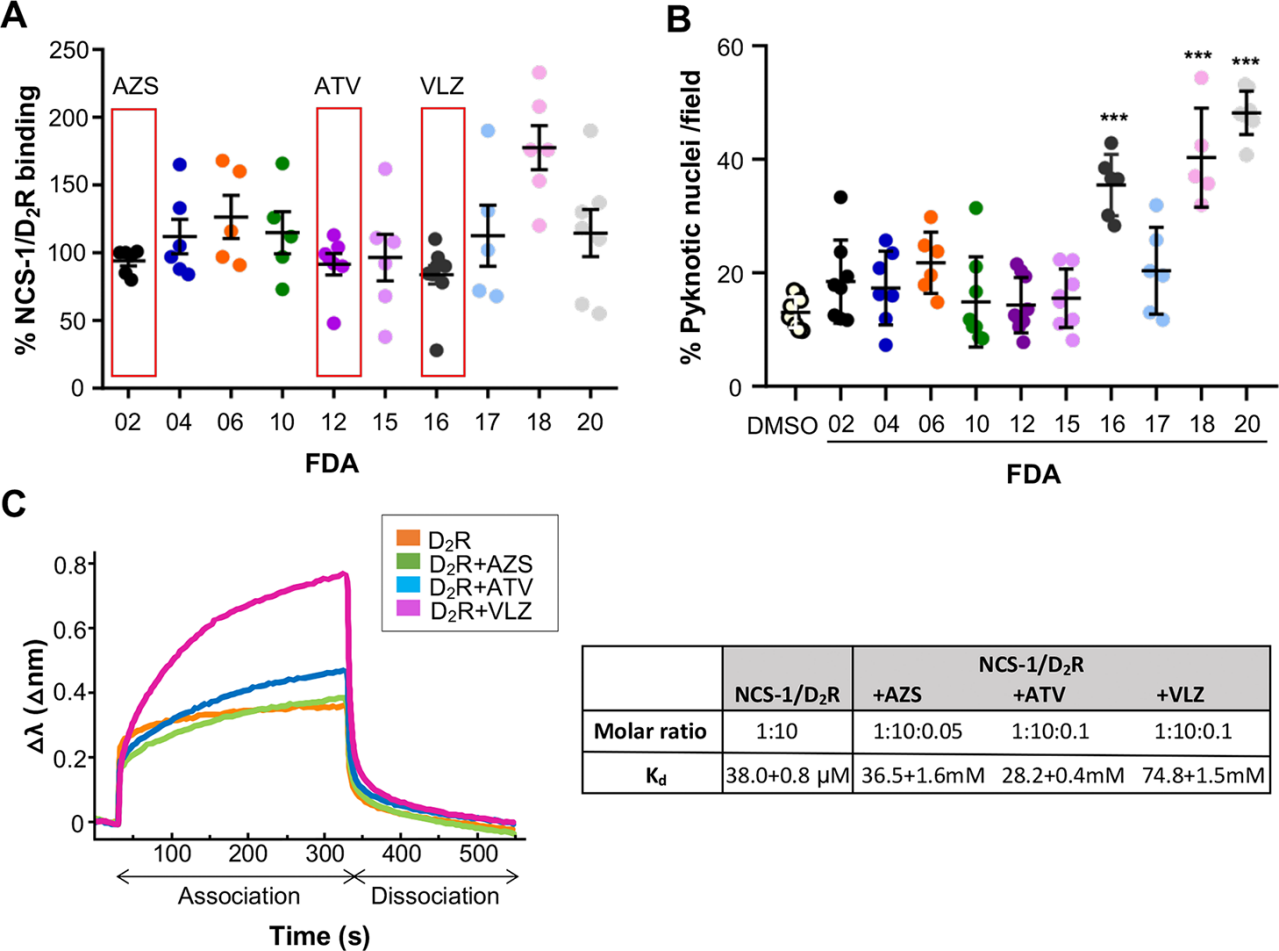
Effect of FDA-approved compounds on NCS-1/D_2_R interaction and cellular toxicity. (A) Proximity ligation assay (PLA) assessing the interaction between NCS-1 and D_2_R in HEK293 cells treated for 16 h with 5 μM of the indicated FDA-approved compounds. Data are expressed as a percentage of NCS-1/D_2_R binding relative to vehicle-treated controls (set at 100%). Each dot represents an independent experiment; horizontal lines indicate the mean ± SD. Red boxes highlight compounds for which the majority of values fall below 100%, indicating potential inhibition of NCS-1/D_2_R PPI. (B) Cytotoxicity analysis of the same compounds (5 μM, 16 h treatment) in HEK293 cells, assessed by the percentage of pyknotic nuclei per field. Data represent 2–3 fields per condition from three independent experiments. DMSO was used as a vehicle control. Bars represent the mean ± SD ***p < 0.001. (C) In vitro binding between His-NCS-1 and a D_2_R H8 peptide (25) in the absence or presence of azilsartan medoxomil (AZS), atorvastatin (ATV), and vilazodone (VLZ). Representative biolayer interferometry sensorgrams showing association and dissociation of the D_2_R peptide over the time. Data are represented as the wavelength shift (Δλ) during the association and dissociation phases. A table summarizing the molar ratio (NCS-1:D_2_R H8:FDA) and the apparent *K*_d_ values (mean ± SEM) between NCS-1 and D_2_R H8 in the absence or presence of ligands is shown.

To exclude cytotoxic effects as a confounding factor, we performed a nuclear morphology-based toxicity. The percentage of pyknotic nuclei was quantified in HEK293 cells treated with the same compounds under identical conditions. The majority of compounds did not show significant cytotoxicity compared to vehicle (DMSO) treated cells. Although cells treated with vilazodone (FDA16) exhibited significantly higher toxicity compared to vehicle-treated cells, cell death did not exceed 50% (Figure 6B). Importantly, additional titration experiments showed that lower, noncytotoxic concentrations of vilazodone retained biological activity (Supplementary Figure 1B,C), supporting the specificity of its effect. Due to its stronger modulatory effect on the NCS-1/D_2_R interaction, further studies were pursued with vilazodone alongside atorvastatin and azilsartan medoxomil.

To further evaluate the modulatory activity of azilsartan medoxomil, atorvastatin and vilazodone on NCS-1/D_2_R PPI, biolayer interferometry (BLI) assays were performed using a peptide containing the D_2_R helix H8.^26^ The affinity of NCS-1 for the D_2_R peptide was analyzed in the presence and absence of these compounds, as the binding of such small molecules are not directly detectable in single-channel BLItz systems. In the absence of FDA-approved drugs, the measured NCS-1/D_2_R affinity was consistent with previous binding studies, showing an apparent affinity of 38 ± 0.8 μM (Figure 6C).^26^ However, the addition of the FDA molecules significantly weakened the PPI, shifting the dissociation constant from the micromolar to the millimolar range (Figure 6C). Since azilsartan is the molecule with higher affinity for NCS-1 (Figure 5), lower amounts of compound are needed to achieve inhibition.

### In Vivo Activity of the FDA-Approved Drugs That Modulate the NCS-1/D_2_R Complex

As we have demonstrated so far, the primary role of NCS-1 is to regulate the amount of D_2_R available at the plasma membrane, a function of critical importance, as it directly influences the activity of the postsynaptic neuron, or of dopaminergic neurons in cases where D_2_R acts as an autoreceptor. Therefore, if the compounds identified and characterized in this work as inhibitors of the NCS-1/D_2_R interaction are effective, they should interfere with the role of NCS-1 to promote D_2_R trafficking to the membrane. To test this hypothesis, D_2_R localization experiments were repeated in cells cotransfected with D_2_R and NCS-1 in the presence of the compounds. The results revealed a complete reversal of the effects induced by NCS-1 overexpression (Figure 7). Notably, vilazodone retained its ability to block NCS-1–induced D_2_R trafficking even at lower, noncytotoxic concentrations (0.5–2 μM), confirming that its effect is independent of cytotoxicity (Supplementary Figure 1B,C). Interestingly, FDA16 also affected D_2_R localization in the absence of NCS-1 overexpression, likely by targeting the interaction between transfected D_2_R and endogenous NCS-1 naturally present in HEK293 cells (Supplementary Figure 1C).To further assess the specificity of this effect, we included FDA07 in the trafficking assay, a compound with lower affinity for NCS-1 (Figure 5A). FDA07 produced a modest effect on D_2_R localization in the context of NCS-1 overexpression, and the magnitude was significantly lower than that of FDA16, supporting a clear correlation between binding affinity and functional impact (Supplementary Figure 1D). These findings reinforce the interpretation that the observed effects are not widespread or nonspecific, but rather depend on the compound’s affinity and ability to disrupt the NCS-1/D_2_R interaction.

**Figure 7:**
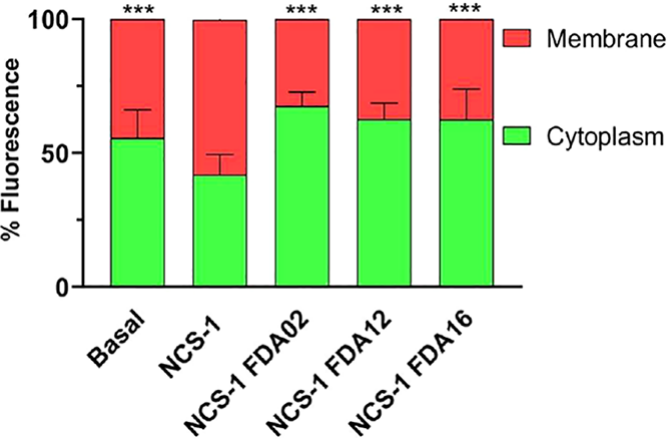
Effect of the compounds on NCS-1-mediated D_2_R cellular localization. Quantification of D_2_R fluorescence distribution between the plasma membrane (red) and cytoplasm (green) in HEK293 cells transfected with D_2_R alone or cotransfected with NCS-1, with or without a 2 h treatment using 5 μM of the indicated FDA-approved drugs. Statistical comparison was performed with cells cotransfected with NCS-1 without compounds. Bars represent the mean ± SD of five fields per condition from three independent experiments. ***p < 0.001.

### Mechanism of Action of the FDA-Approved Drugs That Modulate the NCS-1/D_2_R Complex

To investigate the mechanism by which azilsartan medoxomil (AZS), atorvastatin (ATV), and vilazodone (VLZ) modulate the NCS-1/D_2_R protein–protein interaction, the crystal structures of NCS-1 in complex with these compounds were determined. Crystals were obtained for full-length NCS-1 in complex with ATV and NCS-1ΔH10 in complex with AZS and VLZ (see Experimental Section). The structures were solved by molecular replacement using the structure of NCS-1 (PDB ID: 6QI4,^23^).

#### Structure of the NCS-1/AZS Complex

The asymmetric unit contained a single molecule of NCS-1ΔH10 (Table 1). However, the 2F_o_-F_c_ and the F_o_-F_c_ electron density maps revealed domain swapping between EF-hand motifs, with two NCS-1 molecules exchanging their EF-hand EF-4 (Figure 8A and Supplementary Figure 4A). The resulting NCS-1 structure, comprising EF-hands 1 to 3 from one molecule and EF-hand 4 from a second molecule, closely resembles the canonical monomeric conformation in which EF-hands pair in a two-by-two arrangement without domain swapping. The root-mean-square deviation (RMSD) for Cα atoms is 1.2 Å when compared to the reference NCS-1 structure (PDB ID: 6QI4,^23^).

**Table 1:** Processing and Refinement Statistics of NCS-1/FDA Crystals

NCS-1 complexes	azilsartan medoxomil NCS-1ΔH10/AZS	atorvastatin NCS-1/ATV	vilazodone NCS-1ΔH10/VLZ
PDB code	9GTO	9GU6	9GU8
beamline	ID23-2 (ESRF)	ID30-A3 (ESRF)	XALOC (ALBA)
wavelength (Å)	0.873	0.968	0.979
space group	*C*2_1_	*P*_1_	*C*222_1_
cell dimensions			
*a*, *b*, *c* (Å)	111.11 34.50 55.34	55.29, 59.62, 65.45	44.61 102.91 76.7
α, β, γ (°)	90 95.85 90	87.36, 89.96, 85.06	90 90 90
resolution (Å)	55.26–2.14 (2.35–2.14) [a* = 2.28, b* = 2.06, c* = 2.51]	65.38–1.93 (2.12–1.93) [a* = 1.93, b*=2.24, c* = 2.08]	51.46–1.62 (1.82–1.62) [a* = 1.96, b* = 1.61, c* = 1.83]
*R*_pim_ (%)	0.134 (1.13)	0.193 (0.441)	0.02 (0.55)
CC_1/2_	0.968 (0.164)	0.787 (0.588)	0.999 (0.465)
*I*/σ (I)	4.7 (1.4)	4.3 (1.6)	17.1 (1.3)
completeness			
spherical (%)	70.8 (14.5)	67.8 (13.8)	65.8 (10.9)
ellipsoidal (%)	85.9 (38.2)	84.3 (50)	89.3 (47.5)
multiplicity	3.1 (3.3)	3.1 (2.5)	12.3 (8.9)
refinement			
resolution (Å)	37.15–2.30	24.70–1.93	51.46–1.67
no. of reflections	8141	42456	15142
*R*_work_/*R*_free_	21.27/24.44	21.91/25.60	20.55/23.40
asymmetric unit content			
no. of atoms	2893	12,937	2893
protein molecules	1	4	1
protein residue n°	7–174	6–189, 6–190, 7–189, 7–189	7–175
no. of ligands per protein chain	1 in B	2 in B and D, 1 in F and H	1 in B
Ca^2+^/Na^+^ ions	3/0	12/8	3/2
PEG/GOL/DMSO/ACT molecules	0/0/2/0	0/0/0/5	2/2/0/0
MLI/SIN/FMT molecules	0/0/0	8/10/1	0/0/0
water molecules	75	540	94
B-factor (Å^2^)	35.9	8.4	30.6
R.m.s. deviations protein			
bond lengths (Å)	0.002	0.004	0.005
bond angles (Å)	0.458	0.58	0.64

**Figure 8:**
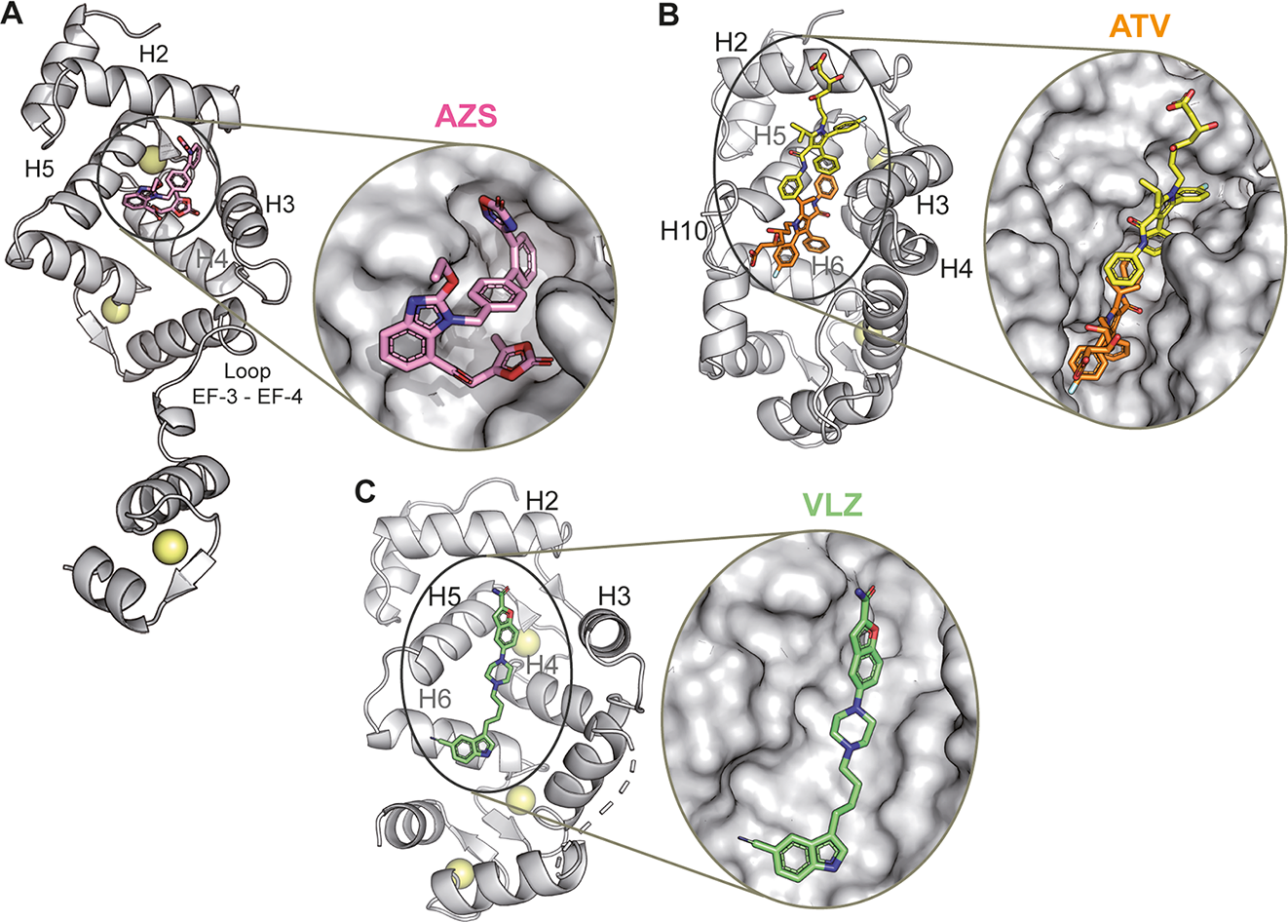
Crystal structures of NCS-1 in complex with the D_2_R PPI inhibitors. (A) Azilsartan medoxomil (AZS, PDB ID: 9GTO); (B) atorvastatin (ATV, PDB ID: 9GU6); and (C) vilazodone (VLZ, PDB ID: 9GU8) are shown in stick mode, NCS-1 in gray ribbons, and Ca^2+^ ions as yellow spheres. A zoom on the ligand binding site is shown. The NCS-1 molecular surface is represented. The NCS-1 helices participating in the recognition are indicated.

A single molecule of AZS was bound to NCS-1, occupying the upper region of the protein’s hydrophobic cavity (Figure 8A and Supplementary Figure 4B,C). The interface between AZS and NCS-1 spans a contact area of 468.8 Å^2^, comprising 490 interactions, predominantly mediated by van der Waals forces. Ligand recognition involves 15 NCS-1 residues located within EF-hand 1 (helices H2 and H3) and EF-hand 2 (helices H4 and H5). The carbonyl oxygen of the AZS ester moiety forms a direct hydrogen bond with Y52, positioned at the base of the cavity. Additionally, AZS establishes two further hydrogen bonds via water-mediated interactions. Notably, all aromatic moieties of AZS, including the benzimidazole, two phenyl rings, oxadiazole, and dioxolane, engage in π-π stacking with surrounding aromatic residues (Figure 8A and Supplementary Figure 4C).

Since NCS-1 crystallized in the presence of AZS as a domain-swapped dimer (possibly due to the concentrations of DMSO in the crystallization solution), molecular dynamics (MD) simulations were performed with monomeric full-length NCS-1 in complex with azilsartan medoxomil. A crystal structure of NCS-1 in which the helix H10 is located outside the hydrophobic cavity was used (PDB ID: 6QI4,^23^). AZS coordinates from the NCS-1/AZS crystal structure (Figure 8A) (PDB ID: 9GTO) were transferred to the full-length NCS-1 structure. Following structural alignment and coordinate transfer, a 1000 ns molecular dynamics (MD) simulation was performed. Throughout the simulation, a rearrangement of helix H10 was observed and after approximately 500 ns, the helix inserted into the hydrophobic crevice (Movie 1) and remained in that conformation for the rest of the simulation, as indicated by the RMSD values of the backbone atoms (Supplementary Figure 5A).

To identify the most representative and stable structure from the MD simulations, a clustering analysis was performed (Supplementary Figure 5B). A total of ten clusters were initially identified using a hierarchical agglomerative (bottom-up) algorithm with average-linkage, which considers the average distance between members of two clusters. Among the resulting clusters, Cluster 0 emerged as the most populated, comprising 42.8% of the total frames (42,764 frames). This cluster exhibited an average intracluster distance of 1.60 Å with a standard deviation of 0.32 Å, indicating a relatively compact conformational ensemble. The centroid of this cluster corresponds to frame 68,966, which was selected as the representative structure for further analyses. Additionally, the average distance of the cluster members to the centroid (AvgCDist) was 2.54 Å, further supporting the centrality of the selected representative conformation.

Cluster 0 appeared after approximately 50,000 frames (corresponding to 500 ns), which coincides with the stabilization of the structure. The superposition of the representative NCS-1/AZS complex of Cluster 0 with the crystal structure yields an RMSD of 1.48 Å, indicating a close agreement between the simulated and experimental binding modes (Figure 9). In this representative structure, a hydrogen bond is formed between a carbonyl oxygen from AZS and the backbone nitrogen from L189, which is located at the end of helix H10 (Figure 9 and Supplementary Figure 6). In addition, a weak hydrogen bond is transiently formed between an oxygen from the AZS oxadiazole group and the F85 side chain. Analysis of the binding site revealed that the ligand is in close proximity to 16 residues, forming van der Waals contacts. These residues are the same as those described in the crystal structure with the exception of L189 (Supplementary Figure 4C).

**Figure 9:**
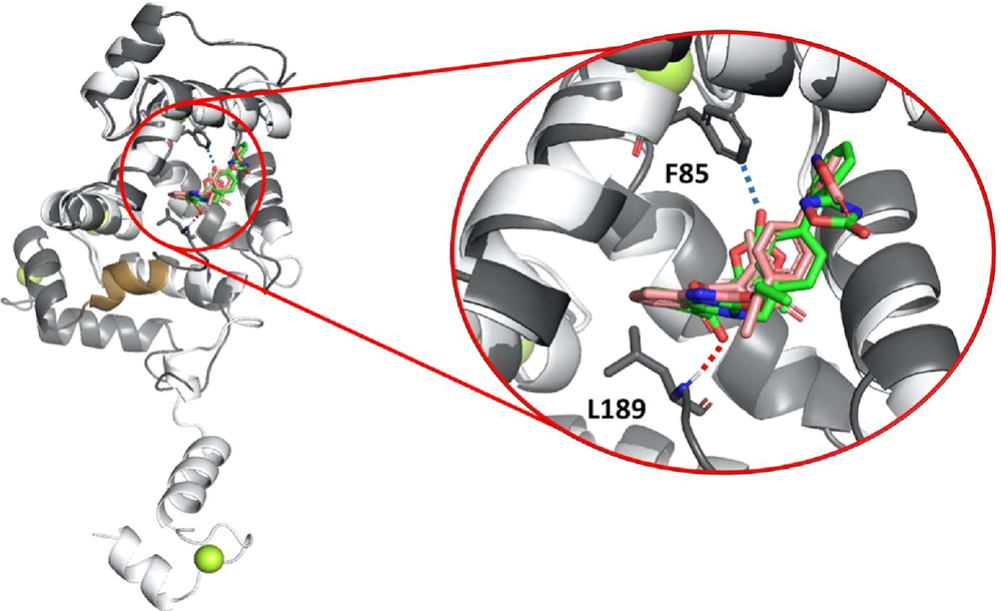
Molecular dynamics simulations of the NCS-1/AZS complex. Superposition of the crystal structure (PDB ID: 9GTO) and the representative structure from cluster 0 obtained from molecular dynamics (MD) simulations. The X-ray crystal structure is shown in light gray (AZS in pink), while the MD-derived structure is depicted in dark gray (ligand in green). Helix H10 is highlighted in gold. A close-up view of the AZS binding mode is also provided.

#### Structure of the NCS-1/ATV Complex

The crystal structure of NCS-1 in complex with atorvastatin revealed four independent NCS-1 molecules in the asymmetric unit (Table 1 and Supplementary Figure 7A–C). The 2Fo–Fc and Fo–Fc electron density maps showed NCS-1:ATV complexes with both 1:1 and 1:2 stoichiometries, where the drug occupied the hydrophobic crevice of the calcium sensor (Figure 8B and Supplementary Figure 7A–C). NCS-1 molecules B and D contained two atorvastatin molecules: the first (ATV1) binds near the entrance of the crevice, while the second (ATV2) is positioned deeper within the cavity (Figure 8B and Supplementary Figure 7A,B). In contrast, NCS-1 molecules F and H contained only one ligand (ATV1), located at the same site as in molecules B and D. Differences among the four NCS-1 molecules are limited to the ligand-binding regions, and within each pair (B/D and F/H), the protein structures are virtually identical, with RMSD values for Cα atoms of 0.151 Å (B vs D) and 0.155 Å (F vs H). Comparisons between molecule D and F or H yields RMSDs of 0.795 or 0.752 Å, respectively.

Within the asymmetric unit, molecules B and F interact through their Ca^2^^+^-binding interfaces, while molecules D and H associate via the opposite face, which includes the hydrophobic crevice (Supplementary Figure 7A). Chains D and H are arranged antiparallel to each other (Supplementary Figure 7B). Notably, in both cases, ATV1 is buried within the protein interface and shielded from solvent by an adjacent NCS-1 molecule, with F58 acting as a lid. In contrast, the second ligand, ATV2, remains solvent-exposed (Supplementary Figure 7B). All ATV1 and ATV2 molecules adopt similar conformations (Supplementary Figure 7D,E). However, weak or discontinuous electron density was observed for the 3,5-dihydroxypentanoyl tail, indicating flexibility and weak interactions with NCS-1. This region displayed the highest conformational variability. The most notable difference among the ATV2 ligands lies in the benzamide group, which interacts with adjacent ATV1 molecules. While variable in chains F and H, this group adopts a stable conformation in chains B and D to interact with ATV2 (Supplementary Figure 7C–E).

Protein–ligand interaction analysis has been made on NCS-1 chain D, where electron density for both ATV ligands was best (Supplementary Figure 7C). Both ligands span from the very top to the middle of the hydrophobic crevice. ATV1 engages 10 NCS-1 residues in helices H2, H3, and H5 (Figure 8B and Supplementary Figure 7F,H), forming 355 contacts across a 423 Å^2^ interface. Binding is dominated by van der Waals interactions, with aromatic π–π stacking playing a major role. The central pyrrole ring of ATV1 is sandwiched between aromatic side chains on both sides of the cavity. Additional hydrophobic contacts further stabilize the complex. The benzamide moiety of ATV1 also interacts with the methylpropane group of ATV2 (Supplementary Figure 7F–I). ATV2 interacts with 13 residues (Supplementary Figure 7G,I). Its central pyrrole ring lies between T92 and Y52, both of which form direct hydrogen bonds with the adjacent amide group. A hydroxyl group on the dihydroxypentanoyl tail forms a water-mediated hydrogen bond with T92 and contacts the C-terminal end of helix H10. Additionally, the pyrrole nitrogen forms an H-bond with a malonate ion present in the crystallization solution. Although ATV2 also establishes van der Waals contacts with aromatic and hydrophobic residues, it shows fewer π–π interactions than ATV1, and the distances between its aromatic moieties is greater (Supplementary Figure 7H–I).

#### Structure of the NCS-1/VLZ Complex

The 2Fo–Fc and Fo–Fc electron density maps revealed a single copy of vilazodone bound within the hydrophobic groove of NCS-1. The ligand is very linear, compared with VLZ and ATV and binds the upper and central regions of the groove (Table 1, Figure 8C and Supplementary Figure 8A). The NCS-1–vilazodone interface spans 420 Å^2^ and involves 396 contacts across 13 NCS-1 residues (Supplementary Figure 8B,C). Vilazodone primarily engages the left side of the cavity, interacting with residues from helices H2, H5, and H6 Figure 8C and Supplementary Figure 8). Additional van der Waals interactions are contributed by the lower part of VLZ, involving helices H4 and H6. A key interaction is a hydrogen bond between Y108, located at the bottom of the binding pocket, and the indole ring of vilazodone. On the opposite side of the cavity, the ligand’s amide group forms a hydrogen bond with a polyethylene glycol (PEG) molecule. In the central region, the nitrogen atom of the piperazine ring forms an H-bond with a water molecule. Furthermore, extensive van der Waals contacts are observed with surrounding hydrophobic and aromatic residues, including notable π–π stacking between the benzofuran moiety of vilazodone and the side chain of F72 (Figure 8C and Supplementary Figure 8).

## Discussion and Conclusions

Targeting protein–protein interactions (PPIs) remains a challenging but promising strategy in drug discovery, particularly for neurological disorders where modulating function without full inhibition can be therapeutically advantageous. Here, we focused on disrupting the interaction between the calcium sensor NCS-1 and the dopamine D_2_ receptor, a PPI that regulates receptor localization without altering its canonical signaling profile. This approach differs fundamentally from direct receptor antagonism, offering a way to influence dopaminergic tone by altering receptor availability at the membrane rather than interfering with ligand binding or G protein coupling. By biasing the receptor toward intracellular pools and away from the presynaptic membrane, dopaminergic neurons can attenuate the autoinhibitory feedback normally mediated by surface D_2_ autoreceptors, thereby sustaining physiologically appropriate dopamine release without changing ligand affinity or downstream signaling capacity. While reducing D_2_R presence at the presynaptic membrane may enhance dopamine release, this redistribution could also affect postsynaptic signaling, particularly in striatal medium spiny neurons (MSNs) expressing D_2_Rs. It is conceivable that any reduction in postsynaptic D_2_R activity could be counterbalanced by increased extracellular dopamine resulting from presynaptic disinhibition. Further in vivo studies will be required to determine the net effect of this shift in receptor distribution on dopaminergic circuit function.

NCS-1 has been proposed to stabilize D_2_R at the plasma membrane by blocking internalization, possibly through inhibition of GRK2-mediated phosphorylation of the D_2_R intracellular C-terminal region and interaction with GRK2.^14^ However, our data suggest a different mechanism. Pharmacological inhibition experiments indicate that NCS-1 does not primarily prevent endocytosis but rather promotes D_2_R surface localization via a Brefeldin A–sensitive exocytic pathway (Figure 2). While further studies are needed to fully resolve this mechanism, our findings point toward a role for NCS-1 in facilitating forward trafficking rather than acting solely as a retention factor. Additionally, overexpression of NCS-1 does not have an impact on the pharmacological profile of the D_2_R in a cellular model system (Figure 3), concluding that NCS-1 likely interacts with D_2_R during trafficking. Although the cellular BRET assays are insensitive to receptor expression and receptor reserve, the change in surface expression in vivo is however likely to enhance D_2_R signaling, where the amount of receptor in the surface and their relative amount to intracellular transducers has an impact on signaling and the cellular response. Hence, the higher amount of receptor at the plasma membrane mediated by NCS-1 is likely to have an impact in a physiological context.

Similarly, D_2_R can homo and heterodimerize with other receptors in native tissues.^35–37^ In fact, NCS-1 binds to D_2_R homodimers and D_2_R-A_2A_R heterodimers.^38^ Hence, NCS-1 could potentially influence D_2_R dimerization and heterodimerization. Due to the highly complex network of D_2_R oligomerization it is hard to predict the overall functional effect. However, since NCS-1 seems to bind D_2_R during trafficking, and D_2_R oligomers are at equilibrium at the plasma membrane,^39,40^ we expect that NCS-1 will allow D_2_R to equilibrate into the necessary oligomeric forms once at the cellular surface. Nevertheless, some oligomeric forms could take place after translation and insertion into the ER membrane and affect trafficking,^41^ hence it cannot be discarded that NCS-1 could affect such early formed oligomers, affecting their native trafficking in cells where those receptors coexpress.

The identification of active molecules through a structure-based virtual screening of FDA-approved drugs (Figure 4) highlights the potential of drug repurposing in neuropharmacology. Among the hits, azilsartan medoxomil, atorvastatin, and vilazodone showed measurable affinity for NCS-1 (Figure 5) and disrupted the NCS-1/D_2_R interaction in vitro and in cells (Figure 6). These compounds, with documented clinical use and varying CNS penetrance, offer attractive starting points for future development, to further improve certain characteristics such as protein affinity, PPI inhibitory activity or brain penetration.

Crystallographic and biophysical data revealed that these molecules bind to the both spacious and conformationally adaptable hydrophobic crevice of NCS-1, overlapping with the D_2_R recognition site and thus competing with D_2_R for NCS-1 binding (Figures 6 and 8). Notably, the ligands exploit a combination of van der Waals contacts, π–π stacking, and hydrogen bonding, providing a diverse interaction footprint across the crevice (Supplementary Figures 4, 7, and 8). Azilsartan medoxomil binds to the upper region of the NCS-1 crevice, and molecular dynamics simulations suggest that its binding does not interfere with the insertion of the dynamic helix H10 into the lower part of the crevice (Figure 9). In contrast, atorvastatin and vilazodone also localize to the upper half of the crevice but extend further into the central region. These small molecules partially overlap with an inserted helix H10 (Supplementary Figure 10), suggesting that the compounds disrupt the dynamics of this critical structural element, thereby hindering its proper insertion into the binding cavity. In fact, the deletion of the helix H10 improves their binding to NCS-1 (Supplementary Figure 9). This disruption of the dynamics of helix H10 may account for the observation that, compared with AZS, both ATV and VLZ exhibit comparable inhibition of the protein–protein interaction despite their lower binding affinities (Figure 5), as ligand-induced conformational rearrangements likely destabilize the D_2_R recognition interface. These structural insights not only validate the mechanism of inhibition but also offer a template for rational optimization of NCS-1-targeted PPI modulators. In fact, although the ligands do not share a common pharmacophore, the interactions between NCS-1 and AZS or ATV are mostly conserved (Supplementary Figures 4, 7, and 8). Both ligands establish a H-bond with Y52 and all aromatic residues and hydrophobic residues that are exposed to the top half of crevice establish π–π stacking or van der Waals contacts with the ligands. In the case of VLZ, the interaction pattern is less conserved with respect AZS and ATV. However, there are several residues that play an important role in the recognition of the three ligands: W30, Y52, F55, F72, F85 and L89, suggesting the relevance of these interactions in ligand recognition and PPI modulation (Supplementary Figures 4, 7, and 10).

Interestingly, other Ca^2+^ binding proteins such as calmodulin (CaM;^42^) and calneuron^38^ also interact with the dopamine D_2_ receptor, despite being structurally dissimilar and recognizing the receptor through distinct mechanisms. CaM binds to the intracellular loop 3 (ICL3) of D_2_R primarily via electrostatic interactions, whereas calneuron engages both ICL3 and helix H8. All these differences suggest that the FDA-approved drugs modulating the interaction of D_2_R and NCS-1 may not have an impact on CaM or calneuron. Likewise, NECAB2, which belongs to a different Ca^2+^ binding family of proteins, is able to recognize and regulate other receptors such as adenosine A_2A_ ^43^ or the metabotropic glutamate receptor type 5,^44^ having an impact on cell surface expression, as is the case of adenosine A_2A_ receptor. In this context, our findings suggest that other protein–protein interactions may be subject to regulation, highlighting the need for high-resolution structural information to enable the rational design of specific modulators of these PPIs.

Collectively, our results reveal a novel therapeutic strategy based on modulating intracellular protein–protein interactions to regulate dopamine D_2_ receptor trafficking. Rather than blocking receptor signaling directly, this approach shifts receptor localization to fine-tune dopaminergic tone, offering a targeted alternative to conventional antagonism. Critically, the identification of FDA-approved compounds as modulators of the NCS-1/D_2_R interaction underscores the untapped potential of drug repurposing in neuropharmacology. While further studies are needed to determine the therapeutic relevance, pharmacological manipulation of receptor trafficking may offer a promising strategy to address dopaminergic dysregulation in conditions such as schizophrenia, bipolar disorder, and Parkinson’s disease.

## Experimental Section

### Virtual Screening of Drug-like Compounds

#### Ligand Preparation

An initial list of 1352 FDA-approved drugs was retrieved from the ZINC database. The conversion from SMILES to SD format was performed using the structconvert tool in the Schrödinger module.^45^ Ligand preparation was carried out using the LigPrep and Epik modules of the Maestro suite.^46–49^ Progressive levels were generated, encompassing possible ionization states at physiological pH and potential tautomers. Final energy minimization was performed using the OPLS4 force field, with default parameters set for stereoisomer.^50^

#### Protein Preparation

NCS-1 proteins, human NCS-1 (hNCS-1, PDB ID: 6QI4,^23^) and Drosophila homologue of human NCS-1 (dNCS-1, PDB ID: 5AAN,^22^) were prepared for subsequent computational analyses using the Protein Preparation Workflow,^51–54^ a tool integrated into Maestro.^49^ This process began with protein structure preprocessing, including bond order assignment and structural adjustments facilitated by Prime.^53,55,56^ Furthermore, PROPKA^57^ was employed to refine the protein’s hydrogen bond network and determine residue protonation states at pH 7.2, leveraging its p*K*_a_ prediction capabilities. Selection of the most favorable protonation state for each was based on hydrogen bond formation and an associated penalty score. The entire preparation concluded with a final restrained minimization utilizing the OPLS4 force field.^50^

#### *S*tructure-Based Virtual Screening (SBVS) and MM-GBSA Rescoring

A set of 2143 molecular candidates from the postprocessed FDA database was screened against the previously described targets. The centroid of the crystallized ligand in the hydrophobic pocket served as the grid center. SBVS was then performed using a pipeline that included 3 stages. The first one consisted of massive docking simulations employing the Glide software^58–61^ and the Standard Precision (SP) method. In this first stage, an enhanced sampling approach was used, and 5 poses were generated per compound state. The top 50% of compounds (according to the scoring function) were retained and used for the second stage, where the Extra Precision (XP) method was employed. In the second stage, 25% of the top-ranked solutions were retained. Rescoring was performed in the third stage with the Prime MM-GBSA method.^53,55,56^

#### Docking Validation

Docking studies were validated by root-mean-square deviation (RMSD) values obtained from the superposition of the redocked ligand structure (IGS1.76) and X-ray crystal structure. The docking grid was centered on the geometric center of the ligand observed in the crystal structure within the catalytic pocket.

### Molecular Dynamics Simulations

The movement of helix H10 toward the crevice and the stability of the X-ray structure NCS-1/AZS was studied using MD simulations with AMBER20.^62^ The NCS-1 structure was prepared using Protein Preparation Workflow,^51–54^ was modified to create zero-order bonds for calcium ions, and all water molecules were removed. The protein model was constructed using the ff14SB force field^63^ with the TIP3P water model.^64^ Three calcium ions were maintained, and neutralizing sodium counterions were added as needed to ensure system neutrality.

Energy minimization was performed to relieve steric clashes and optimize geometry. The protocol consisted of five sequential steps. The first four steps involved positional restraints on the protein backbone with gradually decreasing force constants. These restrained minimization phases employed a hybrid steepest descent/conjugate gradient algorithm, a nonbonded cutoff of 10.0 Å, and a final RMSD convergence criterion of 0.005 Å. Following energy minimization, molecular dynamics (MD) simulations were performed to equilibrate the protein–ligand complex in explicit solvent. The system was thermalized and equilibrated using a series of simulations: initial heating to 100 K with protein restraints (10 ps), followed by further heating to 300 K with restraints (100 ps), and a final 100 ps production run at 300 K without restraints. Temperature and pressure control were achieved using a Langevin thermostat (damping coefficient of 1.0 ps-1)^65,66^ and a Berendsen barostat,^67^ respectively. Bonds involving hydrogen atoms were constrained using the SHAKE algorithm,^68^ and a 10.0 Å cutoff was applied for nonbonded interactions. Production simulations were performed at 300 K under constant pressure and temperature. To generate a 1000 ns trajectory, a 1 ns production protocol was repeated 1000 times. The cpptraj module of AMBER was used for trajectory analysis.^69^

### Molecular Cloning and Constructs

Human NCS-1 constructs, both untagged and V5-tagged, were previously described.^22,70^ The cDNAs of the human D_2_R (HASS-FLAG-EGFP-3C_protease_-D_2_R), MeN (NanoLuc Fragment-membrane anchor) and β-Arrestin-2 (β-Arrestin-2-NanoLuc Fragment) were obtained through gene synthesis (Gene Fragments, Twist Bioscience) and cloned into the pCDNA4 using in vivo DNA assembly.^71,72^ D_2_R was subsequently subcloned into the pCDNA3.1/V5-His-TOPO vector for proximity ligation experiments. The Gα_OA_-RLuc8, Gβ_3_, and Gγ_9_-GFP2 constructs in pCDNA5 and pCDNA3.1 were a gift from Bryan Roth’s lab (Addgene plasmid kit #1000000163). GRK2 (pcDNA4) was a kind gift from Nevin Lambert. The plasmids encoding the human D_2_R linked to the natural peptide and the miniGα_O_ linked to the large-BiT were previously described^73,74^ and kindly provided by Dr. K. Sahlholm (Karolinska Institute, Stockholm, Sweden) and Dr. S. Ferré (National Institutes of Health, Baltimore, MD, USA), respectively.

For biophysical and crystallographic assays, full-length human NCS-1, His-tagged full-length NCS-1 (His-NCS-1), and an helix H10 deletion construct (NCS-1ΔH10) were expressed and purified in Ca^2+^ saturating conditions as published elsewhere.^17,23,75^

### Cell Culture and Transfection

Human embryonic kidney HEK293T cells (ATCC, not authenticated in-house) were grown in DMEM supplemented with 100 U/mL penicillin, 10 mg/mL streptomycin and 10% (v/v) fetal bovine serum. Manipulation and maintenance were done in a biological safety cabinet class 1 and in an incubator at 37 °C, 5% CO_2_ and 90% relative humidity. HEK293T cells were transiently transfected with the indicated cDNA construct using polyethylenimine (PEI MAX, Polysciences Inc.) at a 6:1 PEI:DNA ratio. For D_2_R localization experiments and Proximity ligation assays, transfections were carried out using Lipofectamine 2000 (Invitrogen) in Opti-MEM (Thermo Fisher Scientific) without serum or antibiotics, following the manufacturer’s instructions. After a 4-h incubation with the DNA-Lipofectamine mixture, the medium was replaced with a fresh complete medium, and cells were incubated for an additional 24 h before further processing.

### Immunostaining for D_2_R Localization

HEK293 cells were seeded on poly-d-lysine-coated glass coverslips in 24-well plates. Following transfection and drug treatments, cells were fixed with 2% paraformaldehyde (PFA) for 10 min and with 4% PFA for an additional 10 min at room temperature. Coverslips with fixed cells were then blocked in PBS containing 10% normal goat serum (NGS) for 30 min at room temperature.

To differentiate membrane-localized from cytoplasmic D_2_R, a two-round immunostaining protocol was performed. In the first round, coverslips were incubated with rabbit anti-FLAG primary antibody (Sigma, F7425; 1:1000) for 2 h at room temperature, followed by Alexa Fluor 594-conjugated antirabbit secondary antibody (Thermo Fisher Scientific, 1:500) for 2 h. After three PBS washes, fluorescence was fixed with 10% formaldehyde for 3 min. The second round started with a blocking step using PBS containing 10% NGS and 0.03% Triton X-100 for 1 h at room temperature. Then and incubation with mouse anti-FLAG primary antibody (Sigma, 1:1000) overnight at 4 °C, followed by Alexa Fluor 488-conjugated antimouse secondary antibody (Invitrogen, A10684; 1:500) for 2 h at room temperature. Coverslips were washed with PBS and mounted using ProLong Gold Antifade (Thermo Fisher Scientific) with Hoechst for nuclear counterstaining. Confocal images were acquired using either a Leica SP5 or a Nikon ECLIPSE Ti microscope, depending on the experiment. The analysis of cytoplasmic versus membrane-bound D_2_R was performed using ImageJ in a semiautomated workflow. Individual confocal images were processed by setting intensity thresholds for the green (cytoplasmic) and red (membrane) channels. Fluorescence intensity was then measured separately for each channel, and the relative proportion of green to red signal was calculated for each image.

### Proximity Ligation Assay

HEK293 cells were cotransfected with plasmids encoding human NCS-1 and D_2_R tagged with V5. 24 h post-transfection, cells were treated for 16 h with either FDA-approved compounds or an equivalent volume of DMSO, serving as the vehicle control.

To assess protein–protein interactions, a PLA was performed in conjunction with flow cytometry. PLA enables in situ detection of proteins in close proximity (within 40 nm) by employing antibodies conjugated to DNA oligonucleotides.^34^ Upon binding their respective targets, these probes initiate rolling-circle amplification, producing a fluorescent signal. After treatment, cells were detached using trypsin and gently dissociated then fixed for 20 min using the Fixation Reagent and then permeabilized with the Permeabilization Reagent, both from the Intracellular Fixation and Permeabilization Buffer Set (eBioscience, Thermo), specifically optimized for flow cytometry applications. Primary antibodies; mouse anti-V5 (1:2000, Thermo Fisher Scientific) and rabbit anti-NCS-1 (1:1000, Cell Signaling) were diluted in the permeabilization buffer and added for overnight incubation. Following washes, cells were incubated with Duolink PLA probes: antirabbit MINUS and antimouse PLUS (Sigma). Negative controls omitting the primary antibodies were included to assess background signal. Detection was carried out using the Duolink PLA Flow Cytometry Green Detection Kit (Sigma), according to the manufacturer’s instructions. Fluorescent signals were analyzed using a BD FACS Canto II flow cytometer with BD FACSDiva software.

### β-Arrestin Recruitment Assays

β-arrestin recruitment was assessed in HEK293T cells (ATCC, not authenticated in-house) using the β-arrestin membrane recruitment assay developed by Hauge Pedersen et al.^31^ 50,000 cells/well were seeded in previously poly lysined 96-well white plates. The following day, cells were transfected with D_2_R:MeN:β-Arrestin-2 (ArC):GRK2:pcDNA3.1 or D_2_R:MeN:β-Arrestin-2 (ArC):GRK2:NCS-1 at a 2:1:1:3:4 ratio. After 48 h, the medium was replaced with 90 μL/well of 1× Hank’s balanced salt solution with 20 mM HEPES pH 7.4 and 7.5 μM Coelenterazine 400a. Ten μL of varying quinpirole concentrations were added and luminescence was measured using a CLARIOstar (BMG Labtech) after a 20 min incubation. Data analysis was performed using GraphPad Prism 8.0.1. Data were normalized and a four-parameter logistic curve was fit into the data. Efficacy was normalized relative to D_2_R:pcDNA3.1. Data are presented as mean ± SEM of at least three independent experiments performed in technical triplicate.

### Nanobit MiniGα Protein Recruitment Assay

The NanoBiT assay was performed as previously described.^30^ HEK293T cells were transiently transfected with D_2_R-NP:miniG_O_-LgBiT:pcDNA4.1 or D_2_R-NP:miniG_O_-LgBiT:NCS-1 at a 10:1:20 ratio. After 36 h, cells were harvested in 1× Hank’s balanced salt solution (Cytiva, Utah, USA) and transferred (90 μL) into a white 96 well plate (Corning 96-Well, Cell Culture-Treated, Flat-Bottom microplate; 80,000 cells/cm^2^). Subsequently, 10 μL of a 10 μM coelenterazine 400a solution was added to each well. After 10 min incubation, end-point luminescence was determined using a CLARIOstar plate-reader (basal signal). Immediately after basal measurement increasing concentrations of quinpirole were added and luminescent signal was measured after 10 min. Data was expressed as % of receptor response which was calculated as indicated:

%Receptorresponse=(x−min(x))/(max(x)−min(x))×100

where “*x*” corresponds to the ratio between quinpirole and basal signal of each well and min­(*x*) and max­(*x*) to the ratio of the lowest and highest signal, respectively. Data analysis was performed using GraphPad Prism 8.0.1, with data normalized and fit to a four-parameter logistic curve. Results are presented as mean ± SEM from four independent experiments, each performed in triplicate. Efficacy was normalized relative to D2R:pcDNA3.1.

### Cellular BRET Assays

G protein dissociation was assessed using the TRUPATH system in HEK293T cells.^29^ 50,000 cells/well were seeded in polylysine coated 96-well white plates. The following day, cells were transfected with D_2_R:Gα_OA_-RLuc8:Gβ_3_:Gγ_9_-GFP2:pcDNA3.1 or D_2_R:Gα_OA_-RLuc8:Gβ_3_:Gγ_9_-GFP2:NCS-1 at a 2:1:1:1:4 ratio. After 48 h, the medium was replaced with 90 μL/well of 1× Hank’s balanced salt solution with 20 mM HEPES pH 7.4 and 7.5 μM Coelenterazine 400a. 10 μL of varying quinpirole concentrations were added, and BRET signals were measured using a CLARIOstar (BMG Labtech) with 400 nm (RLuc8) and 498.5 nm (GFP2) emission filters at integration times of 1.85 s. BRET ratios were calculated as the ratio of the GFP2 signal to the Rluc8 signal. Data analysis was performed using GraphPad Prism 8.0.1, with data normalized and fit to a four-parameter logistic curve. Results are presented as mean ± SEM from at least three independent experiments (technical triplicates). Efficacy was normalized relative to D_2_R:pcDNA3.1.

### Ligand Preparation

FDA drugs were purchased to MedChemExpress except in the case of ergotamine, darunavir and dabigatran, which were bought to Sigma. All compounds were >95% pure by HPLC analysis. Compounds were solubilized in pure DMSO at 5 mM (biophysical studies) or 30–50 mM (crystallographic studies) with the exception of amikacin, capastat and hydroxychloroquine, which were soluble in aqueous solution.

### Protein Intrinsic Emission Fluorescence

To determine the affinity of the FDA-approved drugs for full-length NCS-1 or NCS-1ΔH10, we used fluorescence techniques, since the presence of tryptophan (Trp) and tyrosine (Tyr) residues in NCS-1 sequence confers the protein intrinsic emission fluorescence when excited at 295 nm. Fluorescence intensity was monitored at 330 nm and 35 °C with a nano-DSF machine (nanoTemper). Protein concentration was set to 5 μM in a buffer containing Tris 50 mM pH 8.0, NaCl 125 mM, CaCl_2_ 5 μM, 5% DMSO. Ligand concentration was increased up to 150 μM. Three independent experiments were performed for each protein:ligand ratio. Previously, we verified that the compounds did not emit at 330 nm. In fact, compound FDA-02, 09, 11, 15, 21, and 22 were measured using SPR due to their high intrinsic emission at 330 nm. The apparent dissociation constant, *K*_d_, was obtained by using a least-squares algorithm to fit the recorded data to the equation previously described. Apparent *K*_d_ values are reported as mean ± SD.^17,22,33^

### Surface Plasmon Resonance

SPR experiments were performed at 25 °C using a Biacore X-100 apparatus (Biacore, GE) with a running buffer containing Tris 50 mM, pH 8.0, NaCl 125 mM, CaCl_2_ 0.5 mM with 2% DMSO. NCS-1 was immobilized on a CM4 sensor chip (Biacore, GE) following a standard amine coupling method. The carboxymethyl dextran surface of the flow cell 2 was activated by injecting a 1:1 mixture of 0.4 M EDC and 0.1 M NHS for 7 min. The protein was then covalently coupled to the surface via a 7 min injection at several dilutions, with a final concentration of 100 μg/mL in 10 mM sodium acetate, pH 4.0. Unreacted *N*-hydroxysuccinimide esters were quenched by a 7 min injection of 0.1 M ethanolamine-HCl pH 8.0, achieving an immobilization level of 5000 RUs. Flow cell 1, subjected to the same amine coupling procedure but without protein, served as a reference (Supplementary Figure 3A). Prior to use, 10 mM stock solutions of FDA-approved drugs were serially diluted to final concentration of 100 μM in running buffer. Typically, a series of different compounds was injected onto the sensor chip at a flow rate of 30 μL/min for 80s, followed by a 80-s dissociation rate. After dissociation, an additional wash step was performed using a 50% DMSO solution. Regeneration was not required.

For kinetics measurements of FDA02, FDA09, and FDA15, compounds were tested at concentrations ranging from 10 to 100 μM. Compounds were injected at 50 μL/min for a period of 100s followed by a dissociation of 300s. Sensorgrams data were double-referenced and solvent-corrected using the BiaEvaluation X-100 software (Biacore, GE). FDA-21 precipitated on the sensor chip under the experimental conditions and no kinetics measurements were performed.

### Biolayer Interferometry Assay

A single-channel BLItz system (ForteBio) was used to assess the interaction between NCS-1 and a D_2_R H8 peptide,^26^ as well as its modulation by various FDA-approved compounds. This technique immobilizes one of the molecules at the biosensor tip and analyzes interference patterns generated by white light reflection, providing an optical, label-free approach to studying macromolecular interactions. Due to the high concentration of immobilized molecules at the tip, BLI enables the detection of both low- and high-affinity binders, making it particularly suitable for studying weak interactions. Real-time shifts in the interference pattern (Δλ) occur as the number of molecules interacting with those immobilized on the biosensor tip changes. The experimental data obtained from BLI include association and dissociation rate constants, which are used to calculate the apparent equilibrium dissociation constant (*K*_d_).^76^

Ni-NTA biosensors (Sartorius) were used to immobilize the N-terminally His-tagged NCS-1, which was prepared at 5 μM in a buffer A containing 50 mM Tris pH 8, 125 mM NaCl, 128 μM CaCl_2_, and 5% DMSO. The immobilization of His-NCS-1 was performed in three steps: (1) baseline stabilization (buffer A, 30 s), (2) loading (His-NCS-1 in buffer A, 300 s), and (3) equilibration (buffer A, 220 s). D_2_R H8 peptide was prepared at 50 μM in buffer A. In competition assays, the 50 μM D_2_R H8 peptide solution additionally contained 0.25 μM FDA-02 or 0.5 μM FDA-12 and FDA-16. Following protein immobilization, all experiments followed the same sequence: (1′) baseline stabilization (buffer A, 30 s), (2′) association (only D_2_R H8 peptide or its mixture with FDA-approved drugs in buffer A, 300 s), and (3′) dissociation (buffer, 220 s). Each FDA-approved compound was tested in three independent experiments. Control experiments were also performed to discard that neither the peptide or the FDA-approved drugs bound to the biosensor in the absence of protein (Supplementary Figure 3B). The apparent dissociation constant (*K*_d_) was calculated by fitting data extracted from sensorgrams using BLItz Pro software, assuming a 1:1 equilibrium binding model. Apparent *K*_d_ values are reported as mean ± SEM.

### Protein–Ligand Assembly, Crystallization, Diffraction Data Collection, and Structure Solution of NCS-1 Bound to FDA Ligands

Prior crystallization, the contribution of the helix H10 to ligand binding was assessed in case this information could guide construct selection for structural studies. ATV displayed an improvement of the apparent binding affinity for H10-truncated NCS-1 compared with the full-length protein (53 μM vs 17 μM, respectively). Likewise, truncation of the helix H10 also enhanced VLZ binding affinity (34 μM vs 8 μM) (Figure 5C and Supplementary Figure 9). Given the initial high affinity of AZS for full-length NCS-1, the affinity for the H10-truncated construct was not measured. Initial crystallization screenings yielded to crystals of ATV in complex with full-length NCS-1. In the case of AZS and VLZ, the successful construct that yielded to crystals was the truncated NCS-1ΔH10.

Full-length NCS-1 protein was dialyzed in buffer 20 mM NaAc pH 5.5, 0.5 mM CaCl_2_, 0.5 mM DTT and 5% DMSO (2 changes, 4 and 16 h). Similarly, NCS-1ΔH10 protein was dialyzed in buffer 50 mM Tris pH 8.0, 1.75 mM CaCl_2_, 1 mM DTT and 10% DMSO. The proteins were concentrated to 15 mg/mL using a 10 kDa cutoff concentrator (Vivaspin) before ligand addition. AZS and VLZ were added to NCS-1ΔH10 in a 1:2 (protein:ligand) molar ratio, reaching a final DMSO concentration of 15 and 10%, respectively. ATV was mixed with purified full-length NCS-1 in a 1:4 molar ratio, reaching a final DMSO concentration of 5%. Finally, the protein/ligand complexes were set up for crystallization experiments.

Crystals were obtained by the cocrystallization method. After 30 min of protein:ligand incubation, crystallization screenings were set with an Oryx8 robot (Douglas Instruments) at 4 °C, using the sitting drop vapor diffusion method and mixing equal volumes of protein complex and precipitant. NCS-1ΔH10/VLZ complex crystallization were set up at 18 °C. Initial crystals were obtained in solutions from JBScreen 0 at 4 °C. NCS-1ΔH10/VLZ crystals grew in 20% PEG 3000, 0.1 M HEPES pH 7.5, 0.2 M NaAc precipitant solution. Only NCS-1/VLZ crystals were cryo-protected by adding 20% (v/v) glycerol to the crystallization solution. All crystals were flash-frozen in *N*_2_(*l*).

An NCS-1ΔH10/VLZ diffraction data set was collected at 100 K and 0.979 Å wavelength at ALBA BL13 beamline synchrotron radiation source. NCS-1ΔH10/AZS and NCS-1/ATV diffraction data were collected at 0.873 Å wavelength at ESRF ID23–2 and ID30-A3 beamlines, respectively (Table 1). Data were processed automatically at the beamlines with AutoPROC.^77^ Structures were solved by the molecular replacement method with Phaser.^78^ The structures of hNCS-1 (PDB ID: 6QI4,^23^) with or without the C-terminal helix H10 were used as search models, depending on the NCS-1 construct used for crystallization. FDA ligands dictionaries with geometric restrains were generated with acedrg.^79^ Successive cycles of automatic refinement with Phenix^80^ and manual building with Coot^81^ were performed. The final models were validated with Molprobity.^82^ Details on data processing and refinement are shown in Table 1. The structures were analyzed using different programs from the CCP4 package,^83^ LigPlot^84^ and PISA server.^85^ Images were prepared with PyMOL.^86^ The final structures were deposited in the PDB with codes: NCS-1/AZS (9GTO), NCS-1/ATV (9GU6), NCS-1/VLZ (9GU8).

## Supplementary Material









## Notes

Figures, tables, and chromatograms showing the purity of the active FDA-approved drugs used in this study (PDF)
Molecular formula strings, purity, and Cat. No. of purchased FDA-approved drugs and the associated biochemical and biological data (CSV)
PDB files of the 3D docking models of NCS-1 complexes with the FDA-approved drugs selected in this study and ranked in Figure 4 (ZIP)
Movie on the molecular dynamics simulations of monomeric full-length NCS-1 in complex with azilsartan medoxomil (MP4)

## Abbreviations

A_2_AR: adenosine A_2_A receptor
AMBER: assisted model building with energy refinement
AMBER20: 2020 release of AMBER
ArC: β-arrestin C-terminal fragment
ATV: atorvastatin
AZS: azilsartan medoxomil
BFA: brefeldin A
BLI: biolayer interferometry
BRET: bioluminescence resonance energy transfer
BRET2: second-generation BRET assay
CaM: calmodulin
CNS: central nervous system
cpptraj: AMBER trajectory analysis program
D_2_R: dopamine D_2_ receptor
NCS-1ΔH10: NCS-1 construct lacking helix H10
DMEM: Dulbecco’s modified eagle medium
EF-hand: Ca2^+^-binding helix–loop–helix motif
EGFP: enhanced green fluorescent protein
ER: endoplasmic reticulum
ESRF: European synchrotron radiation facility
FACS: fluorescence-activated cell sorting
FMT: formate ion
GEF: guanine nucleotide exchange factor
GFP2: green fluorescent protein variant
Gi/o: inhibitory G-protein families
Glide SP: standard precision glide docking
Glide XP: extra precision glide docking
GOL: glycerol
GRK2: G-protein-coupled receptor kinase 2
H8: intracellular helix 8
H10: helix 10 of NCS-1
HEK293: human embryonic kidney 293 cells
HEK293T: HEK293 cells with SV40 T antigen
HEPES: 4-(2-hydroxyethyl)-1-piperazineethanesulfonic acid
ICL3: intracellular loop 3
InsP_3_R: inositol 1,4,5-trisphosphate receptor
*K*_d_: dissociation constant
LgBiT: large NanoLuc fragment
MeN: membrane-anchored NanoLuc fragment
miniGα: engineered mini Gα proteins
MLI: malonate ion
MM-GBSA: molecular mechanics/generalized Born surface area
MSNs: medium spiny neurons
NanoBiT: NanoLuc binary technology
NanoLuc: engineered luciferase
nanoDSF: nano differential scanning fluorimetry
NCS-1: neuronal calcium sensor 1
NECAB2: neuronal calcium-binding protein 2
Ni-NTA: nickel–nitrilotriacetic acid
NGS: normal goat serum
OPLS4: optimized potentials for liquid simulations force field, version 4
Opti-MEM: optimized minimum essential medium
PFA: paraformaldehyde
PISA: protein interfaces, surfaces, and assemblies
PLA: proximity ligation assay
pEC50: negative base-10 logarithm of EC50
PROPKA: protein p*K*_a_ prediction software
RLuc8: Renilla luciferase 8
Ric-8A: resistance to inhibitors of cholinesterase 8A
SD: standard deviation
SEM: standard error of the mean
SHAKE: bond-constraint algorithm in MD
SIN: succinic acid
SPR: surface plasmon resonance
TIP3P: three-point transferable intermolecular potential water model
TRUPATH: open-source BRET-based GPCR signaling platform
V5: V5 epitope tag
VLZ: vilazodone
XALOC: macromolecular crystallography beamline at ALBA synchrotron
