# Supplementary material for: FDA Drug Repurposing Uncovers Modulators of Dopamine D2 Receptor Localization via Disruption of the NCS‑1 Interaction
Source: J Med Chem. 2025 Nov 10;68(22):23993–4010. doi: 10.1021/acs.jmedchem.5c01626 (PMC12670427; doi:10.1021/acs.jmedchem.5c01626)
Supplement: Supplementary file 1 [file jm5c01626_si_001.pdf]

Supporting Information

FDA drug repurposing uncovers modulators of dopamine D_2 receptor localization via disruption of the NCS-1 interaction.

Daniel Muñoz-Reyes[1], Lorena Aguado[2], Sandra Arroyo-Urea[3], Carlos Requena[4], Sara Pérez-Suárez[1], Sonia Sánchez-Yepes[2], Josep Argerich[3], Celia Miró-Rodríguez[1], Eugenia Ulzurrun[4,5], Eulalia Rodríguez-Martín[2], Javier García-Nafría[3], Nuria E. Campillo[4]*, Alicia Mansilla[2]*, María José Sánchez-Barrena[1]*

[1]Department of Crystallography and Structural Biology. Institute of Physical-Chemistry "Blas Cabrera", CSIC, Serrano 119, Madrid 28006, Spain.

[2]Department of Neurobiology, Instituto Ramón y Cajal de Investigación Sanitaria, Hospital Universitario Ramón y Cajal, Madrid 28034, Spain.

[3]Institute for Biocomputation and Physics of Complex Systems (BIFI) and Laboratorio de Microscopías Avanzadas (LMA), University of Zaragoza, Zaragoza 50018, Spain.

[4]Centro de Investigaciones Biológicas Margarita Salas - CSIC, C/ Ramiro de Maeztu 9, 28040 Madrid, Spain.

[5]Instituto de Ciencias Matemáticas-CSIC, C/ Nicolas Cabrera 13-15, 28049 Madrid, Spain.

*To whom correspondence may be addressed. E-mail: xmjose@iqf.csic.es, alicia.mansilla@uah.es and nuria.campillo@csic.es

List of supplementary material

Supplementary figures and tables:

Supplementary Figure 1: D_2R cellular localization control assays. *S2*
Supplementary Figure 2: Nanobit recruitment assay for the dopamine D_2 receptor in the presence of NCS-1 .... *S3*
Supplementary Figure 3: Control experiments in SPR and BLI assays *S4*
Supplementary Figure 4: Structural details on the complex of NCS-1 with azilsartan medoxomil .. *S5*
Supplementary Figure 5: Molecular dynamics simulations of the NCS-1/AZS complex *S6*
Supplementary Figure 6: Hydrogen bond distances between AZS and L189 or F85 along the molecular dynamics simulations *S7*
Supplementary Figure 7: Structural details of the complex of NCS-1 with atorvastatin *S8*
Supplementary Figure 8: Structural details of the complex between NCS-1 and vilazodone *S9*
Supplementary Figure 9: The binding of atorvastatin and vilazodone to NCS-1ΔH10 .... *S10*
Supplementary Figure 10: Structural comparison of NCS-1/FDA vs NCS-1/D_2R complexes *S11*
Supplementary Table 1: List of the top 20 FDA-approved drugs identified by virtual screenings . *S13*

Purity of active FDA-approved drugs used in this work... *S22*

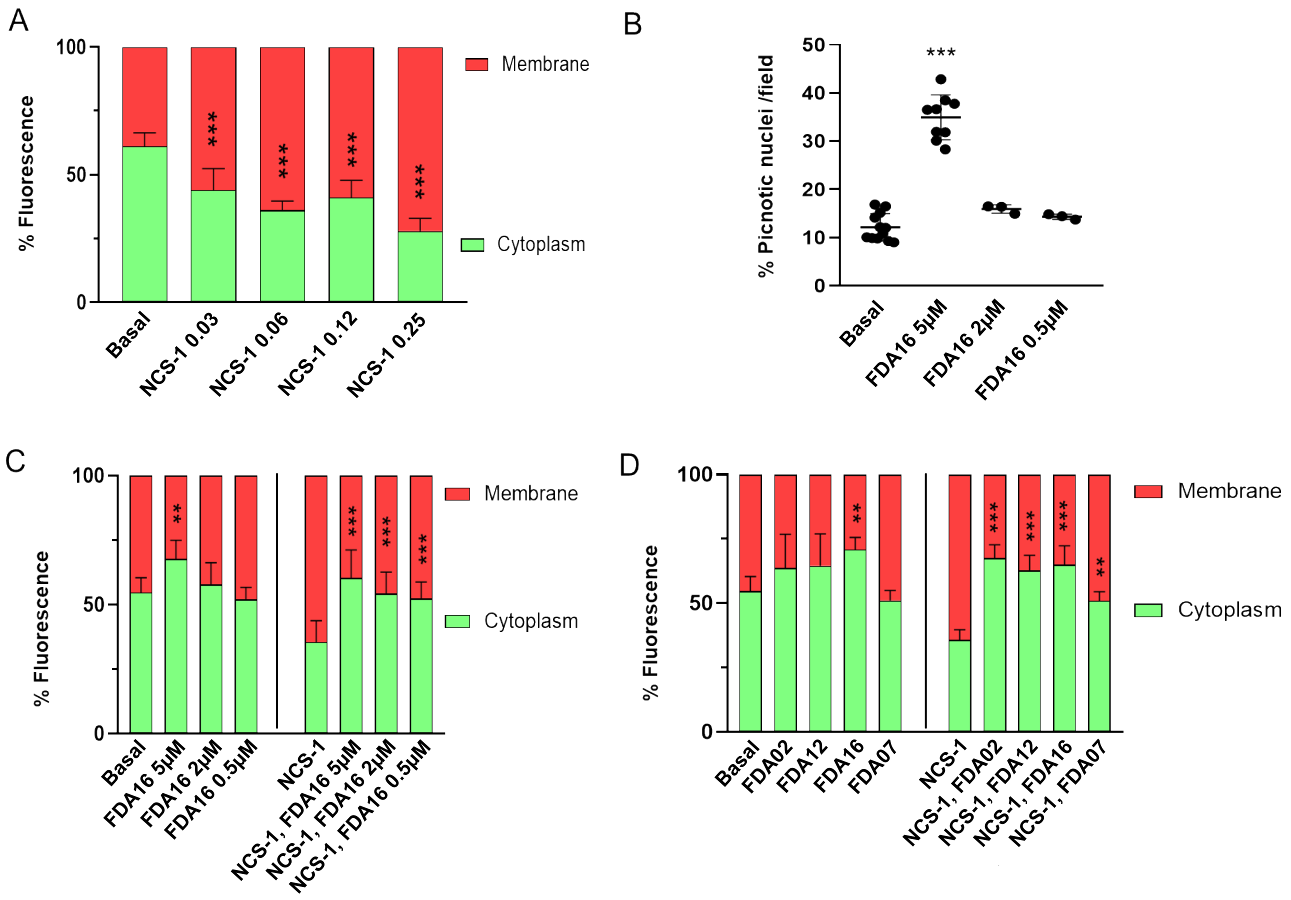

Supplementary Figure 1: D_2R cellular localization control assays. (A) D_2R localization at increasing NCS-1 expression levels. HEK293T cells were transfected with a constant amount of D_2R and varying amounts of NCS-1 to achieve D_2R:NCS-1 ratios of 1:0.03, 1:0.06, 1:0.12, and 1:0.25. **(B)** Cytotoxicity analysis of FDA16 (vilazodone) at 5, 2, and 0.5 μM after 16 hours of treatment in HEK293 cells, measured by the percentage of pyknotic nuclei. DMSO was used as vehicle control. **(C)** D_2R localization in the presence of FDA16 at 5, 2, and 0.5 μM, with or without NCS-1 overexpression. Left panel: statistical comparison is relative to basal condition (D_2R alone); right panel: comparison is relative to NCS-1 overexpressed with D_2R. **(D)** D_2R localization under basal conditions or with NCS-1 overexpression, in the presence of various FDA-approved drugs at 5 μM. Left panel: statistical comparison is relative to basal condition; right panel: relative to NCS-1 overexpression. Bars represent mean ± SD. **$p < 0.01$, ***$p < 0.001$.

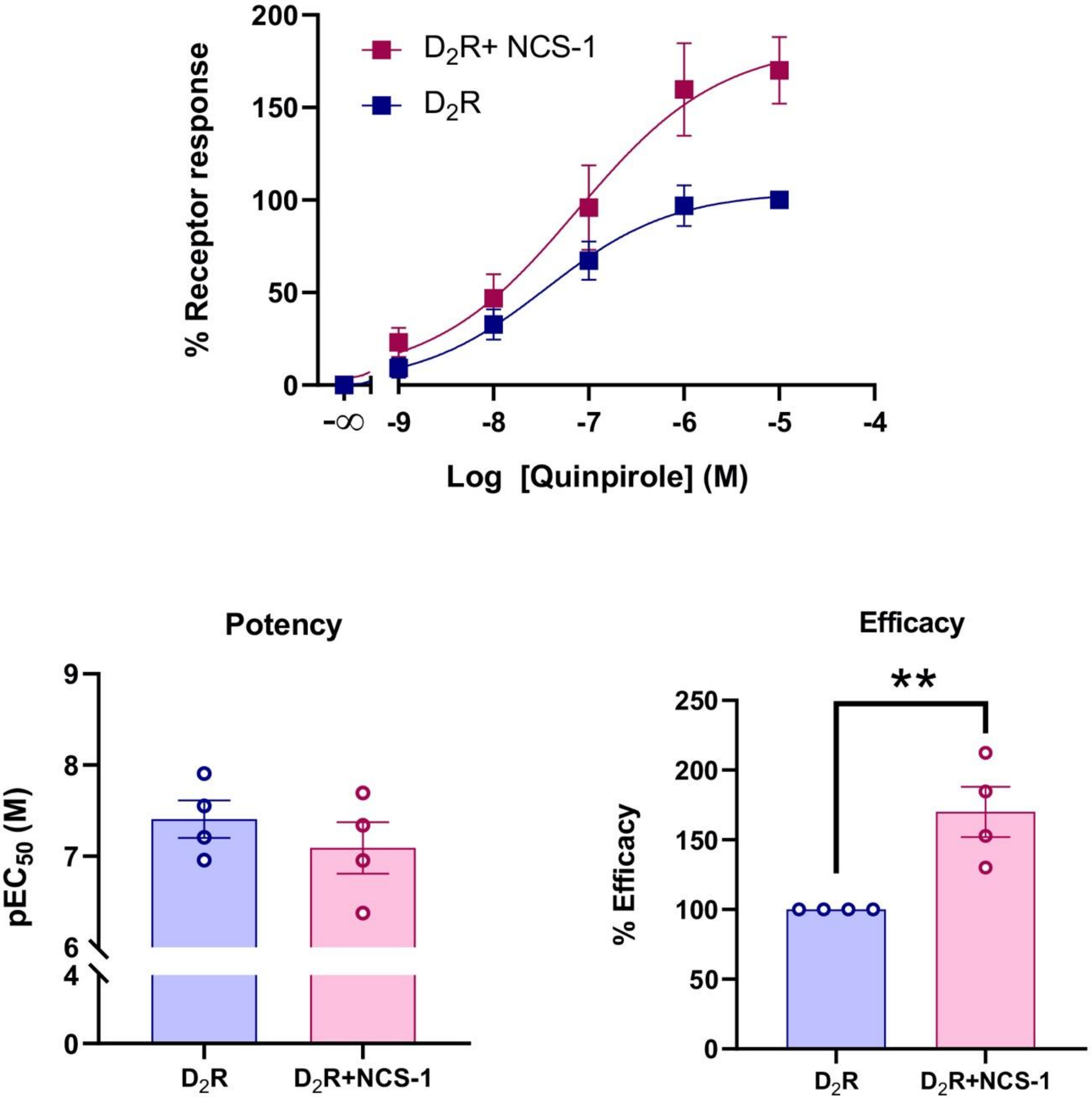

Supplementary Figure 2: Nanobit recruitment assay for the dopamine D_2 receptor in the presence of NCS-1. HEK293T cells expressing D_2R linked to the natural peptide and MiniGαo-LgBiT in the absence (blue squares) or presence (red squares) of NCS-1 were challenged with increasing concentrations of quinpirole and the receptor/MiniGα$_O$ protein coupling was determined by the NanoBiT assay. Luminescence data are expressed as % of receptor response (*see Materials and Methods*). Potency and efficacy of quinpirole dose-response curves in the absence or presence of NCS-1 were assessed. Results are expressed as mean ± SEM of four independent experiments each performed in triplicate. **p<0.01, student's t test.

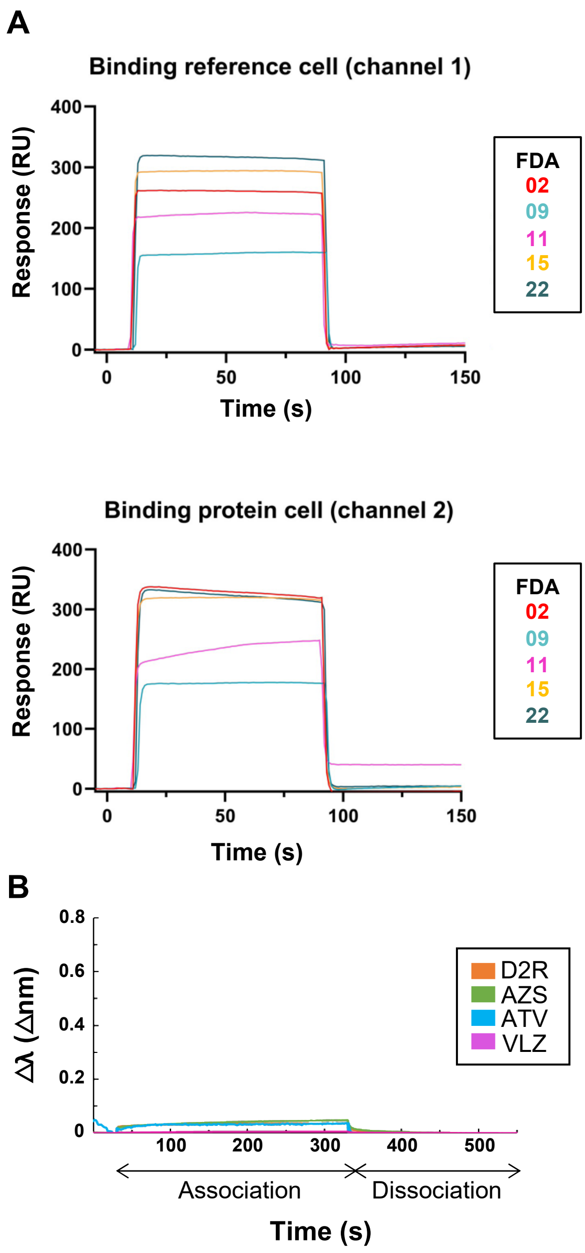

Supplementary Figure 3: Control experiments in SPR and BLI assays. **(A)** Registered sensorgrams of the reference channel 1 (sensor chip without protein) and protein channel 2 (NCS-1 bound to sensor chip) when FDA-approved drugs are injected. **(B)** Recorded sensorgrams when the biosensor is free of NCS-1 and D_2R peptide or FDA-approved drugs are added.

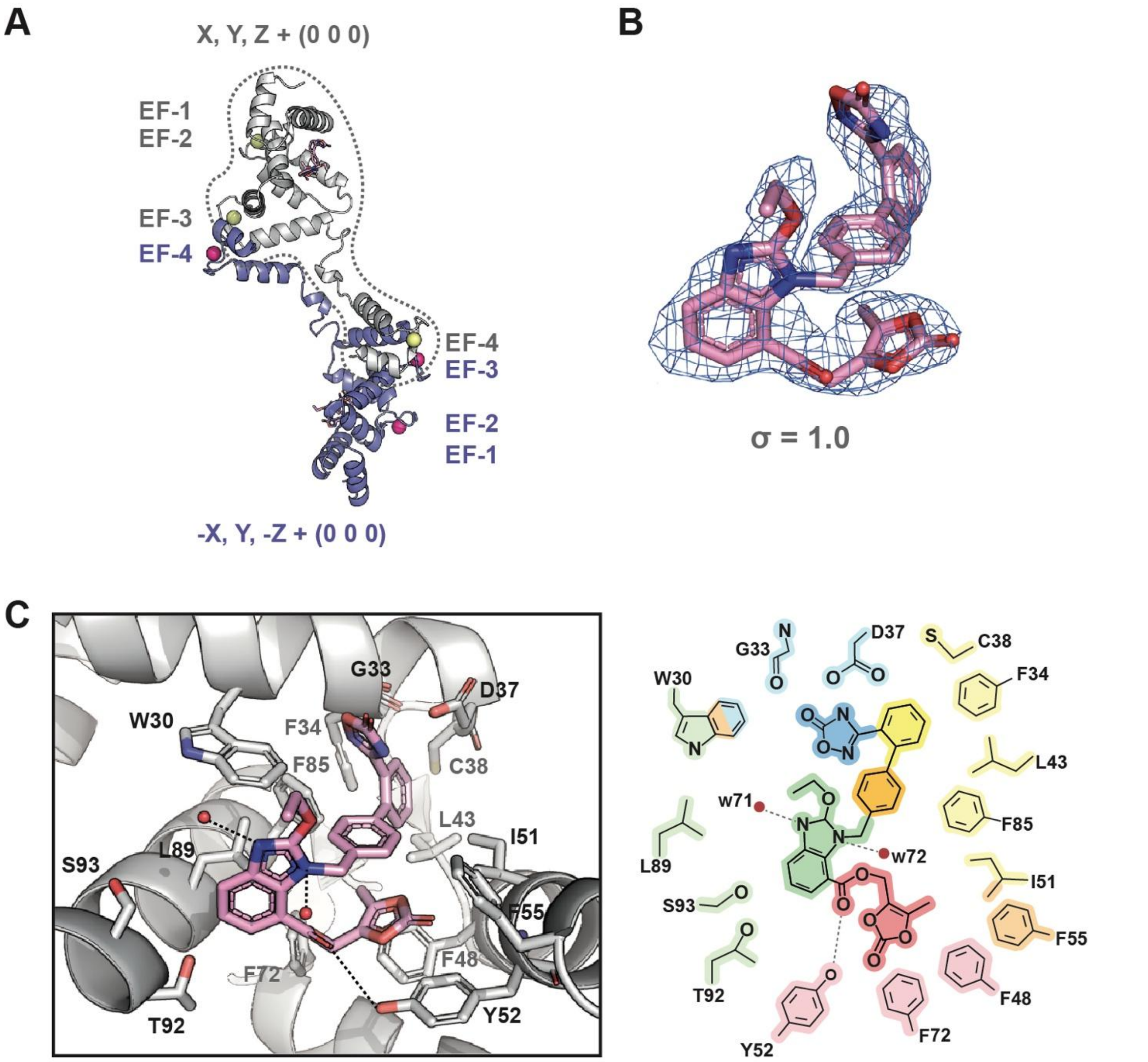

Supplementary Figure 4: Structural details on the complex of NCS-1 with azilsartan medoxomil. (A) Representation of the domain swapping found. The NCS-1 molecule found in the A.U. (PDB ID: 9GTO) is shown in grey and the symmetry related molecule with which the EF-4 motif is exchanged, in blue. **(B)** 2Fo-Fc electron density map. **(C)** Detail of the residues involved in AZS recognition. Hydrogen bonds between NCS-1 (grey) and AZS (pink) are represented as black dashed lines. Water molecules are shown as red spheres. Two-dimensional interaction diagram of the NCS-1/AZS complex. Residues are color-coded according to their interaction with different ligand moieties: oxadiazole (blue), phenyl rings (yellow and orange), benzimidazole (green), ester group and dioxolane (red).

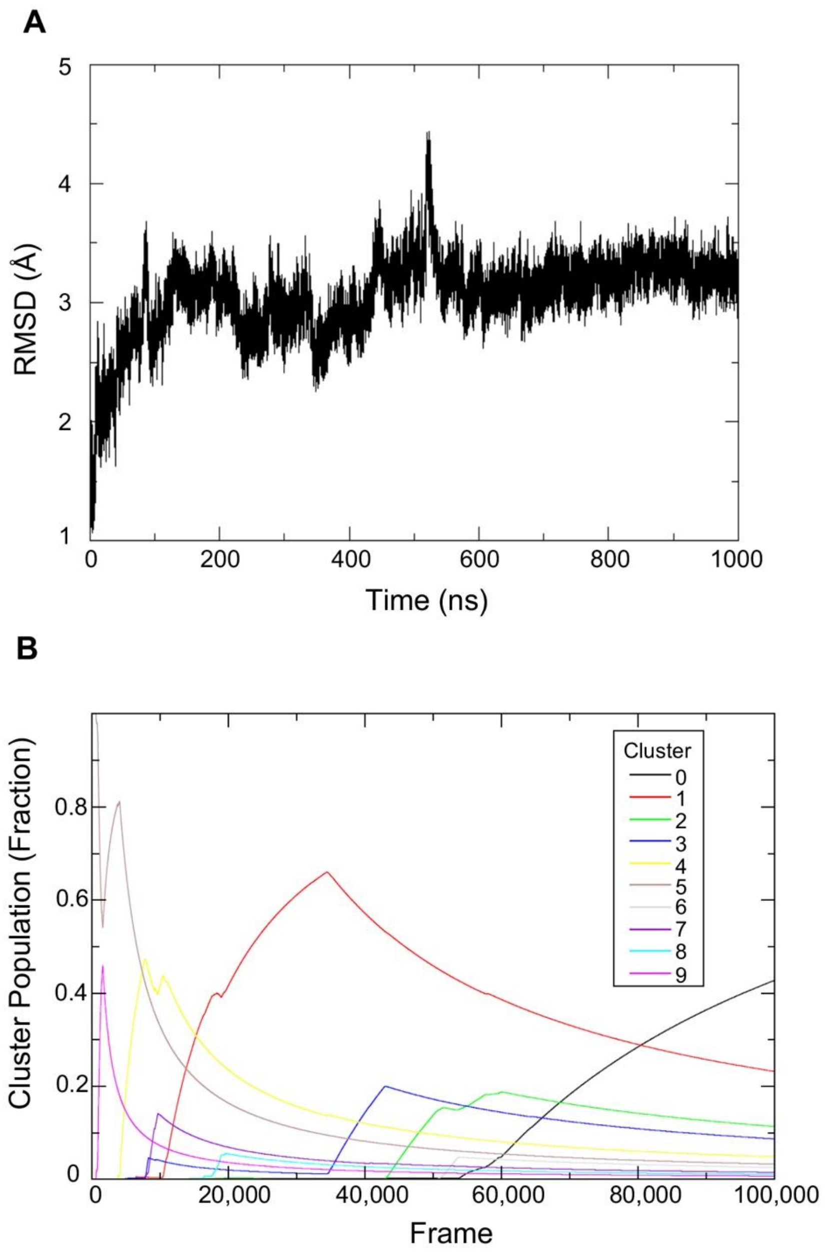

Supplementary Figure 5: Molecular dynamics simulations of the NCS-1/AZS complex. (A) Root-mean-square deviation (RMSD) analysis of the protein backbone atoms (PDB ID: 9GTO) **(B)** Hierarchical clustering of the MD trajectories using the average-linkage algorithm. The energy-minimized structure was used as the reference for aligning the MD trajectories prior to RMSD analysis.

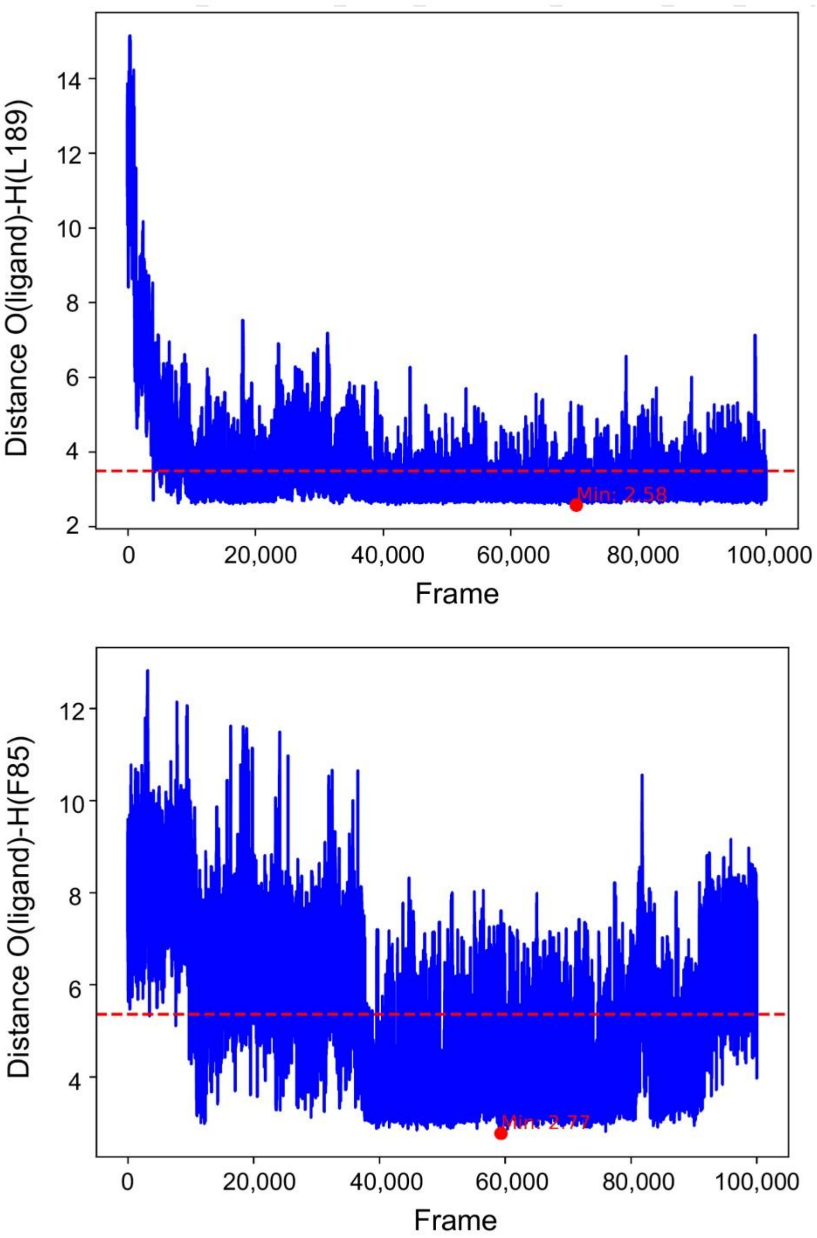

Supplementary Figure 6: Hydrogen bond distances between AZS and L189 or F85 along the molecular dynamics simulations. The mean value is represented by the dashed line, and the minimum value is also indicated. PDB ID: 9GTO.

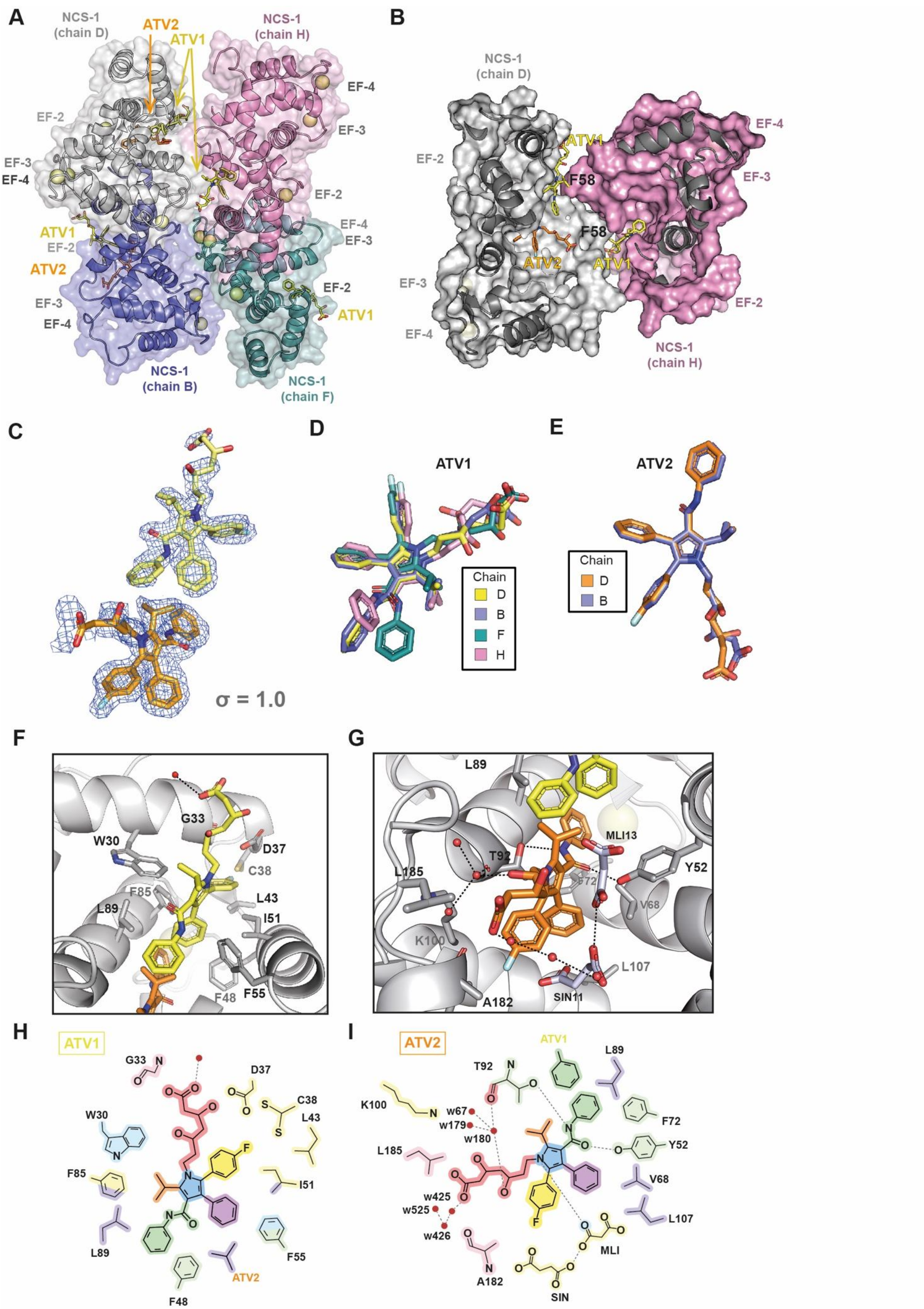

A
NCS-1 (chain D)
ATV2
ATV1
NCS-1 (chain H)
EF-2
EF-3
EF-4
ATV1
ATV2
NCS-1 (chain B)
NCS-1 (chain F)
B
F58
C
σ = 1.0
D
ATV1
Chain
D
B
F
H
E
ATV2
Chain
D
B
F
G33
W30
D37
C38
F85
L43
L89
I51
F48
F55
G
L89
MLI13
T92
L185
Y52
F72
V68
K100
L107
A182
SIN11
H
ATV1
G33
D37
C38
W30
L43
F85
I51
L89
F55
ATV2
F48
I
ATV2
ATV1
L89
T92
K100
w67
w179
w180
F72
Y52
L185
V68
w425
w525
w426
L107
MLI
A182
SIN

Supplementary Figure 7: Structural details of the complex of NCS-1 with atorvastatin. **(A)-(E)** Analysis of the independent molecules comprising the asymmetric unit of the NCS-1/ATV crystal (PDB ID: 9GU6). **(A)** NCS-1 is shown as ribbons and colored according to chain ID: B (purple), D (gray), F (green), and H (pink). The ATV1 and ATV2 ligands are indicated and represented as sticks in yellow and orange, respectively. Ca^{2+} ions are shown as yellow spheres. **(B)** Molecular surface and ribbon representation of NCS-1 chains D (gray) and H (pink). The positions of F58 residues, which act as lids over the ATV1 binding site, are indicated. **(C)** 2Fo-Fc electron density map corresponding to the two ligands (ATV1 and ATV2) bound to NCS-1 chain D, contoured at 1.0 σ. **(D)** and **(E)** Superposition of the ATV ligands bound to NCS-1 and modelled in the A.U. **(F)**, **(G)** Close-up of the residues involved in ATV recognition. Hydrogen bonds between NCS-1 (gray), ATV1 (yellow), and ATV2 (orange) are shown as black dashed lines. Water molecules are represented as red spheres. Malonate (MLI13) and succinate (SIN11) molecules involved in recognition are also depicted. **(H)**, **(I)** Two-dimensional schematic of the interactions between NCS-1 and ATV1 **(H)** and ATV2 **(I)**, respectively. Residues are color-coded according to their interaction with the different ligand groups: 3,5-dihydroxypentanoyl end (red), methylpropane (orange), fluorobenzene (yellow), phenyl (purple), benzamide (green), and pyrrole (blue).

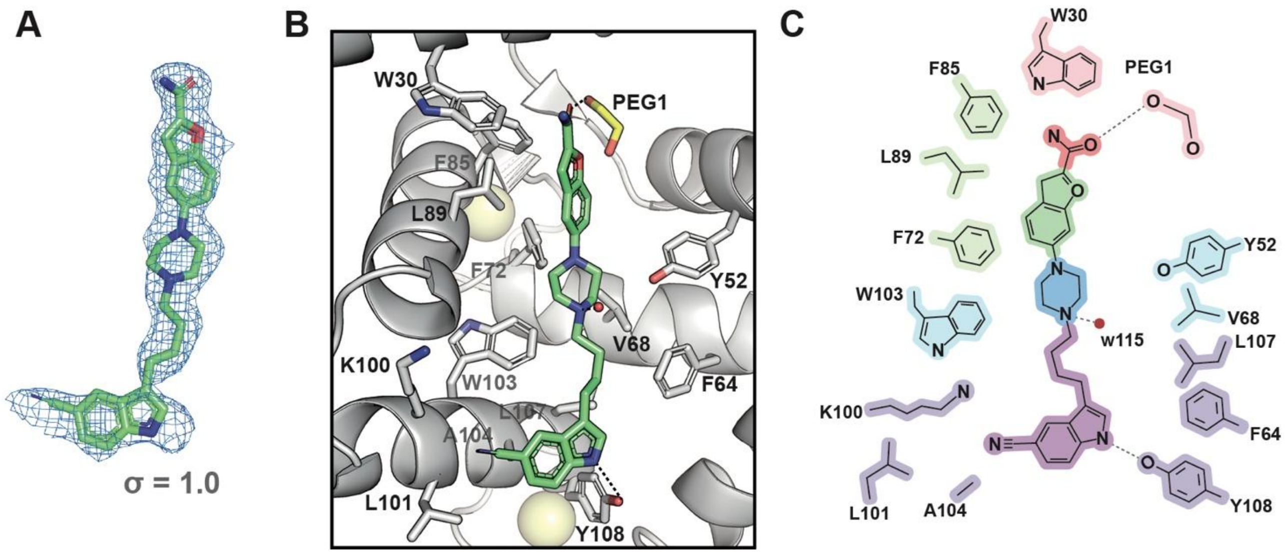

Supplementary Figure 8: Structural details of the complex between NCS-1 and vilazodone. (A) 2Fo-Fc electron density map corresponding to VLZ and contoured at 1.0 σ. **(B)** Interactions between NCS-1 (gray) and VLZ (green) (PDB ID: 9GU8). The residues involved in ligand recognition are shown. Water molecules and Ca^{2+} ions are represented as red and yellow spheres, respectively, and the PEG molecule is shown as yellow sticks. Hydrogen bonds between NCS-1 and VLZ are indicated with black dashed lines. **(C)** Two-dimensional interaction diagram. Residues are color-coded according to their interaction with the different ligand groups: amide (red), benzofuran (green), piperazine (blue), and the aliphatic chain, indole, and cyano groups (purple).

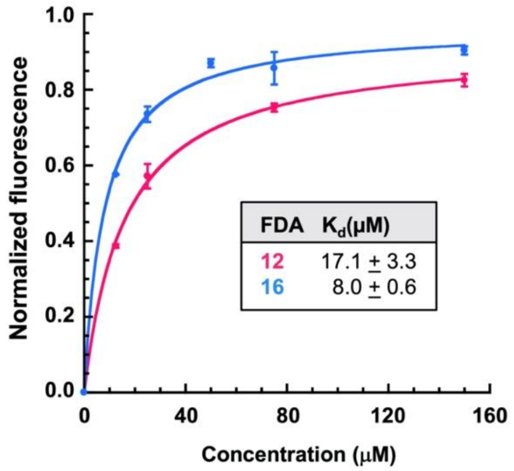

Supplementary Figure 9: The binding of atorvastatin and vilazodone to NCS-1ΔH10. Intrinsic emission fluorescence assays at increasing concentrations of the FDA-approved drugs.

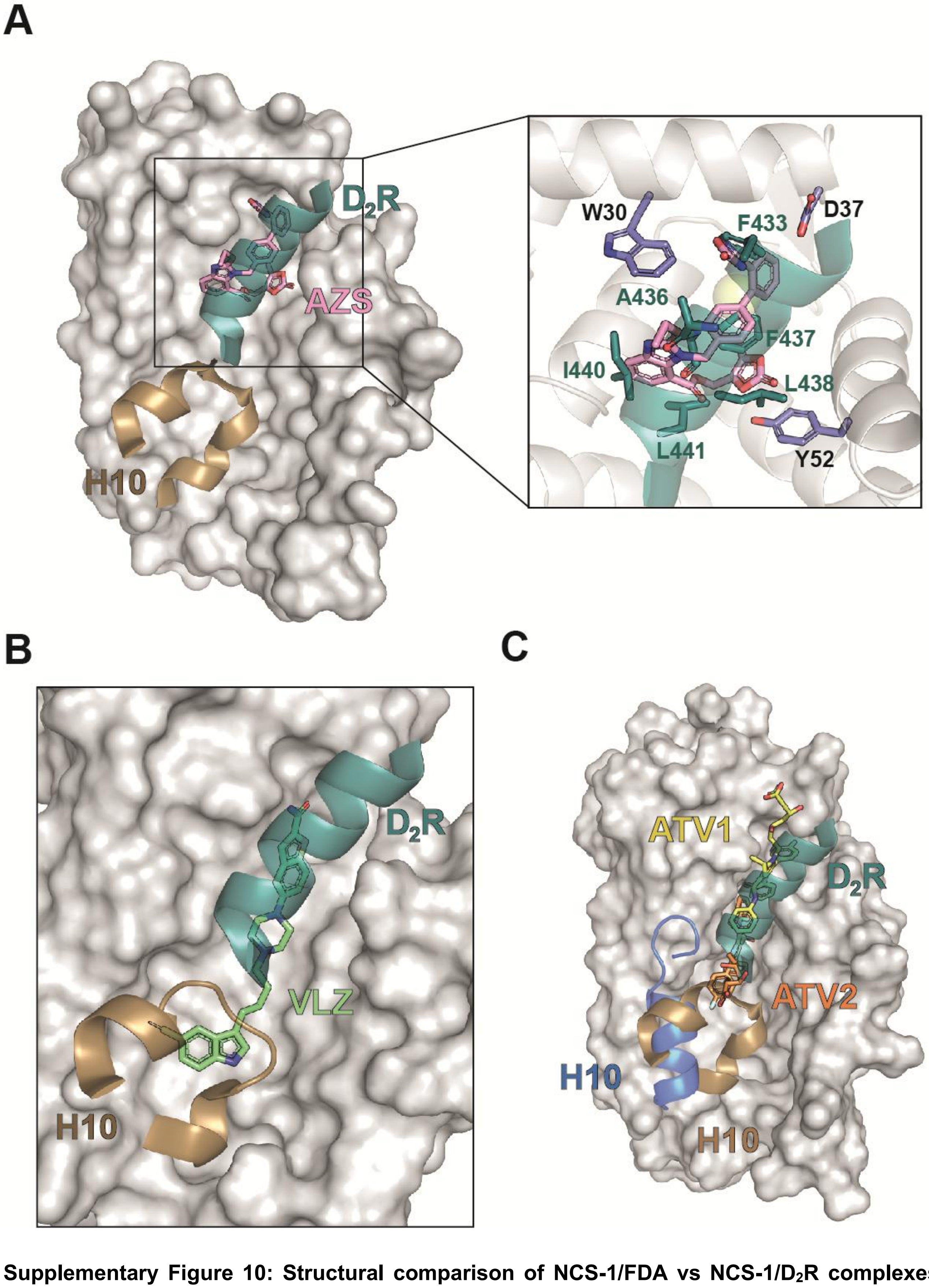

Supplementary Figure 10: Structural comparison of NCS-1/FDA vs NCS-1/D$_2$R complexes. The NCS-1/FDA complexes were superposed onto the NCS-1/D$_2$R H8 complex (PDB ID: 5AER). The NCS-1/D$_2$R H8 complex is depicted as follows: NCS-1 as molecular surface or grey ribbons

(with helix H10 highlighted in gold). Only The D_2R helix H8 (green ribbon) that is placed in the upper part of the cavity is shown, since FDA drugs overlap only with this helix. In the case of the NCS-1/FDA structures, only the FDA-approved drugs (sticks and color code as in Figure 8) and the NCS-1 helix H10 (if present, blue ribbon) are shown. **(A)** The NCS-1/AZS complex. A close-up view highlights how AZS positions its functional groups in regions where the D_2R helix H8 places side chains (shown as green sticks) to mediate recognition by NCS-1 (interacting residues shown as lilac sticks). **(B)** and **(C)** The NCS-1/VLZ and NCS-1/ATV complexes, respectively.

Supplementary Table 1: List of the top 20 FDA-approved drugs identified by virtual screenings. The original therapeutic indication, key residue interactions, docking scores, and MMGBSA binding energies are shown. Dockings were performed with two NCS-1 structural models with PDB ID 5AAN and 6QI4.

Compound FDA-number	Target / Treatment / Crossing the blood-brain barrier	Interactions	QPlogPo/w	QPlogS
**Azilsartan FDA-02**	Angiotensin II type 1 receptor antagonist / Antihypertensive / Low penetrance	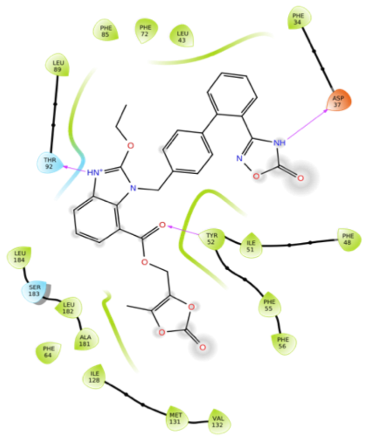	3.851	-7.175
**Sunitinib FDA-03**	Multitargeted tyrosine kinase inhibitor (TKI) / Anticancer, kidney cancer / No	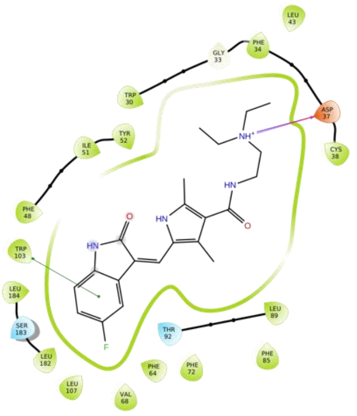	3.781	-4.795

**Nelfinavir FDA-04**	HIV-1 protease inhibitor / HIV antiretroviral / No	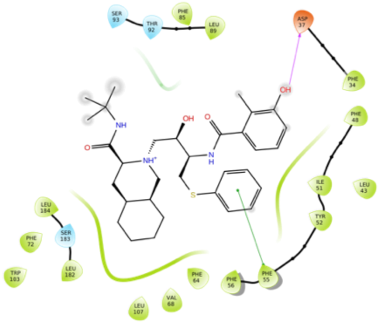	4.443	-6.219
**Ibutilide FDA-05**	Calcium channels / Antiarrhythmic / No	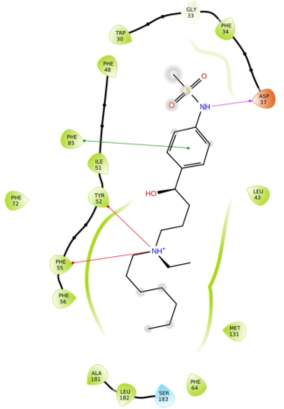	3.242	-1.821
**Lapatinib FDA-06**	Tyrosine kinase inhibitor / Anticancer, breast cancer / No	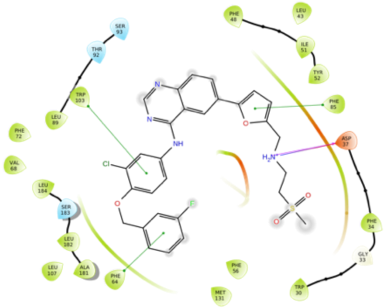	5.901	-6.953

**Ergotamine FDA-08**	5-HT adrenergic and serotonin receptor agonist / Vasoconstrictor, migraines / No	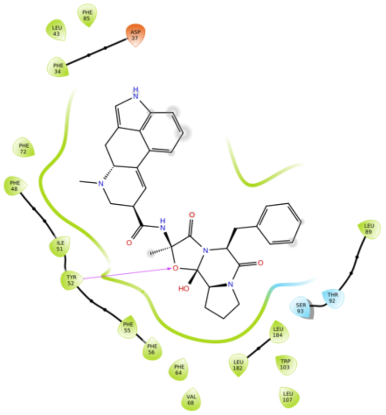	2.435	-2.728
**Darunavir FDA-09**	HIV-1 protease inhibitor / Antiretroviral for HIV / No	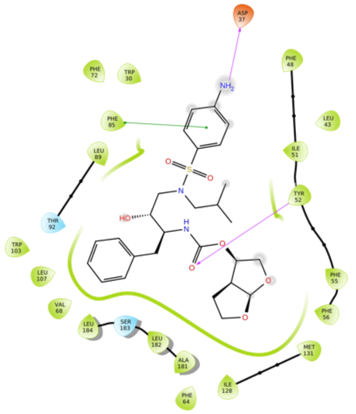	2.69	-4.185

**Dabigatran FDA-10**	Prothrombin, liver carboxylesterase-1, cocaine esterase, UDP-glucuronosyltransferase / Anticoagulant / No	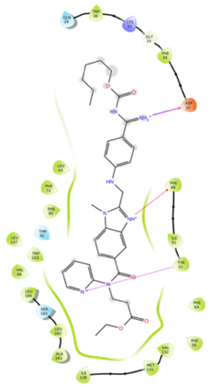	6.348	-9.256
**Faslodex FDA-11**	Estrogen receptor antagonist / Anticancer, breast cancer / No	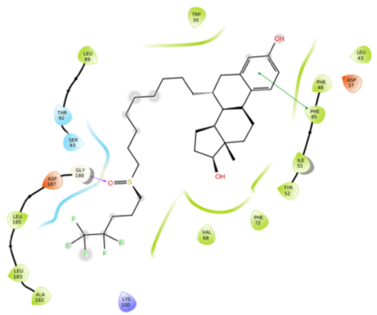	8.037	-8.963
**Atorvastatin FDA-12**	HMG-CoA reductase inhibitor / Dyslipidemia and cardiovascular disease prevention / Yes	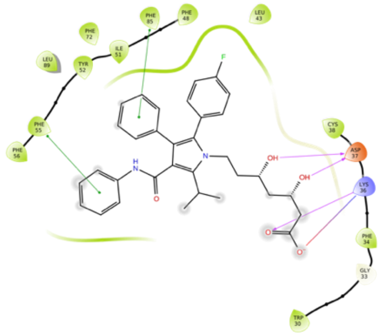	6.901	-7.872

**Hydroxy-cloroquine FDA-13**	RNA polymerase inhibitor; Toll-like receptors (TLRs) / Antimalarial and antirheumatic / Yes	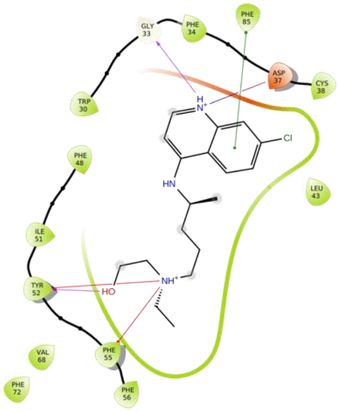	3.312	-3.607
**Cloroquine FDA-14**	RNA polymerase inhibitor; Toll-like receptors (TLRs) / Antimalarial and antirheumatic / Yes	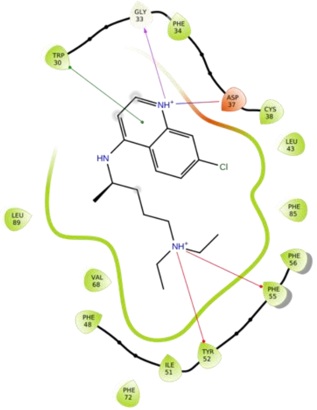	4.399	-4.271

**Salmeterol FDA-15**	Ab2 adrenergic receptor agonist / Asthma /Yes	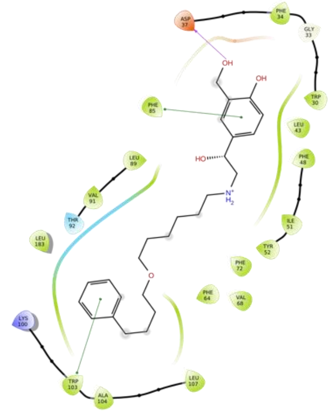	3.732	-3.718
**Vilazodone FDA-16**	Partial agonist of the serotonin 5-HT1A receptor and serotonin transporter inhibitor / Antidepressant / Yes	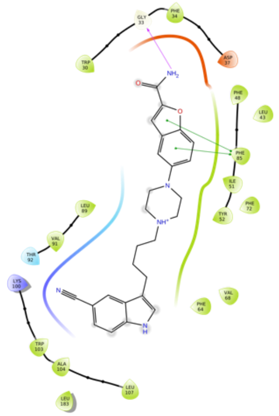	3.023	-6.661

**Clomifene FDA-17**	Estrogen receptor modulator / Female infertility / No	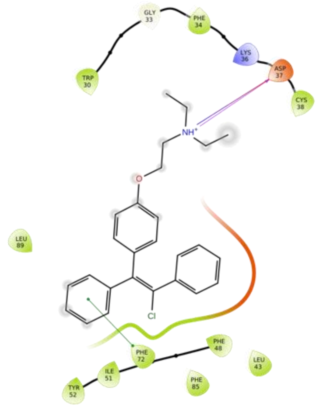	7.006	-6.282
**Ponatinib FDA-18**	Third-generation multitargeted tyrosine kinase inhibitor / Chronic myeloid leukemia / Yes	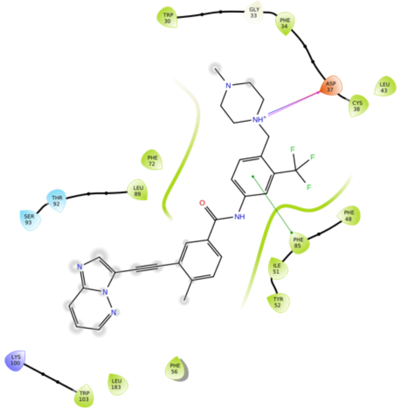	4.37	-6.041

**Imatinib FDA-19**	First-generation tyrosine kinase inhibitor / Chronic myeloid leukemia / Yes	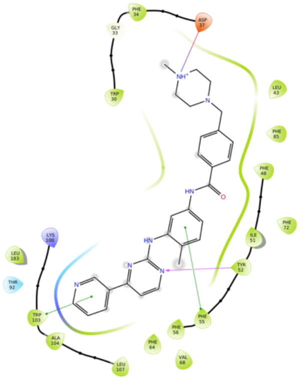	3.679	-5.253
**Daclatasvir FDA-20**	Nonstructural protein 5A (NS5A) inhibitor / Antiviral, Hepatitis C virus / No	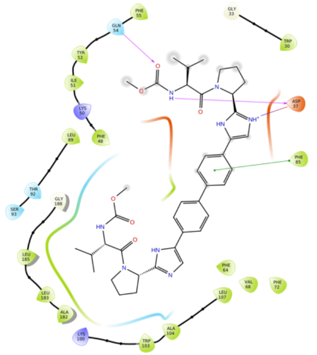	5.814	-9.964
**Protriptyline FDA-22**	Acetylcholinesterase inhibitor / Antidepressant / Yes	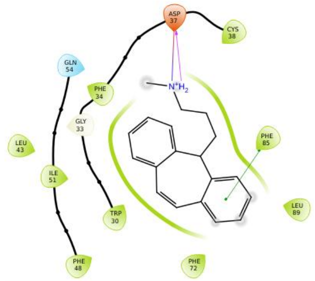	4.791	-4.249

**Fluoxetine FDA-82**	Selective serotonin reuptake inhibitor / Antidepressant, bulimia, or bipolar disorder / Yes	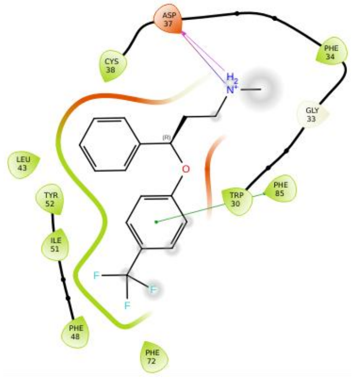	4.46	-3.653

Purity of active FDA-approved drugs used in this work. HPLC traces of azilsartan medoxomil (left upper panel), atorvastatin (right panel) and vilazodone (left lower panel)

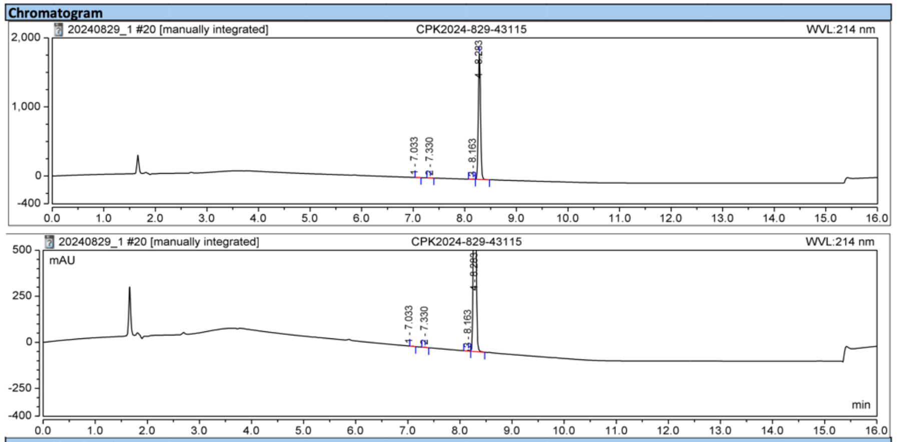

Integration Results

No.	Peak Name	Retention Time min	Area mAU*s	Height mAU	Relative Area %	Resolution (SM)	Plates (EP)	Asymmetry (EP)
1		7.033	2.9866	0.000	0.0551	3.94	n.a.	n.a.
2		7.330	4.6570	1.577	0.0859	11.12	146386	1.14
3		8.163	13.5512	4.741	0.2499	1.53	182807	n.a.
4		8.283	5400.4925	1816.439	99.6091	n.a.	183537	1.05
**Total:**			**5421.6873**	**1822.757**	**100.0000**		**512730**	

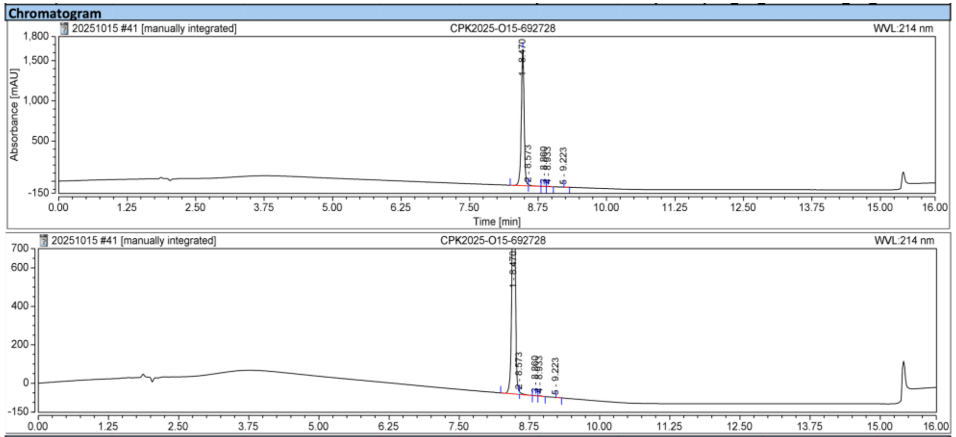

Integration Results

No.	Peak Name	Retention Time min	Area mAU*s	Height mAU	Relative Area %	Plates (EP)	Asymmetry (EP)	S/N
1		8.470	6006.4769	1698.299	98.9648	145998	0.95	3824.6
2		8.573	45.0377	13.940	0.7421	n.a.	n.a.	n.a.
3		8.860	7.3767	1.630	0.1215	n.a.	n.a.	n.a.
4		8.933	9.0361	2.488	0.1489	n.a.	n.a.	n.a.
5		9.223	1.3805	0.000	0.0227	n.a.	n.a.	n.a.
**Total:**			**6069.3080**	**1716.356**	**100.0000**		**1**	

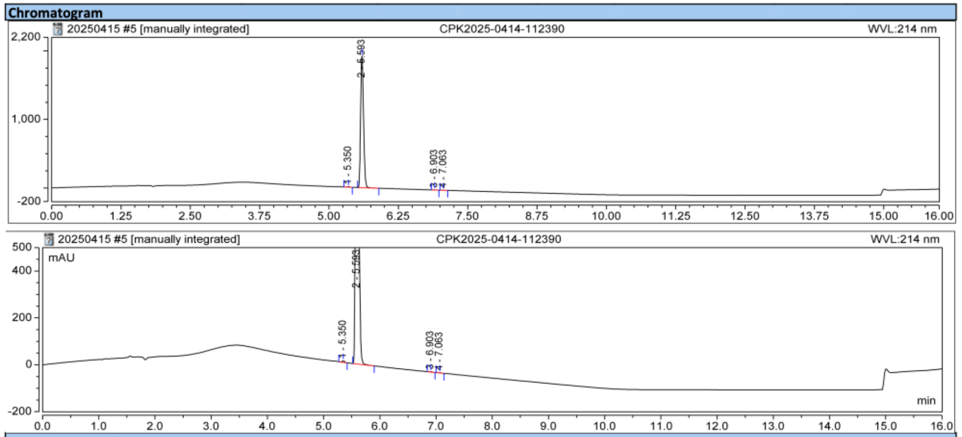

Integration Results

No.	Peak Name	Retention Time min	Area mAU*s	Height mAU	Relative Area %	Resolution (SM)	Plates (EP)	Asymmetry (EP)
1		5.350	19.1682	6.608	0.2749	2.71	76666	1.07
2		5.593	6941.8443	1924.274	99.5673	14.40	54759	1.21
3		6.903	6.1903	1.940	0.0888	2.04	107669	1.24
4		7.063	4.8122	1.572	0.0690	n.a.	122577	1.12
**Total:**			**6972.0149**	**1934.394**	**100.0000**		**361671**	
